# *Coccidioides* Endospores and Spherules Draw Strong Chemotactic, Adhesive, and Phagocytic Responses by Individual Human Neutrophils

**DOI:** 10.1371/journal.pone.0129522

**Published:** 2015-06-12

**Authors:** Cheng-Yuk Lee, George R. Thompson III, Christine J. Hastey, Gregory C. Hodge, Jennine M. Lunetta, Demosthenes Pappagianis, Volkmar Heinrich

**Affiliations:** 1 Department of Biomedical Engineering, University of California Davis, Davis, California, United States of America; 2 Department of Medical Microbiology and Immunology, Coccidioidomycosis Serology Laboratory, University of California Davis, Davis, California, United States of America; 3 Department of Internal Medicine, Division of Infectious Diseases, University of California Davis Medical Center, Sacramento, California, United States of America; Leibniz Institute for Natural Products Research and Infection Biology- Hans Knoell Institute, GERMANY

## Abstract

*Coccidioides* spp. are dimorphic pathogenic fungi whose parasitic forms cause coccidioidomycosis (Valley fever) in mammalian hosts. We use an innovative interdisciplinary approach to analyze one-on-one encounters between human neutrophils and two forms of *Coccidioides posadasii*. To examine the mechanisms by which the innate immune system coordinates different stages of the host response to fungal pathogens, we dissect the immune-cell response into chemotaxis, adhesion, and phagocytosis. Our single-cell technique reveals a surprisingly strong response by initially quiescent neutrophils to close encounters with *C*. *posadasii*, both from a distance (by complement-mediated chemotaxis) as well as upon contact (by serum-dependent adhesion and phagocytosis). This response closely resembles neutrophil interactions with *Candida albicans* and zymosan particles, and is significantly stronger than the neutrophil responses to *Cryptococcus neoformans*, *Aspergillus fumigatus*, and *Rhizopus oryzae* under identical conditions. The vigorous *in vitro* neutrophil response suggests that *C*. *posadasii* evades *in vivo* recognition by neutrophils through suppression of long-range mobilization and recruitment of the immune cells. This observation elucidates an important paradigm of the recognition of microbes, i.e., that intact immunotaxis comprises an intricate spatiotemporal hierarchy of distinct chemotactic processes. Moreover, in contrast to earlier reports, human neutrophils exhibit vigorous chemotaxis toward, and frustrated phagocytosis of, the large spherules of *C*. *posadasii* under physiological-like conditions. Finally, neutrophils from healthy donors and patients with chronic coccidioidomycosis display subtle differences in their responses to antibody-coated beads, even though the patient cells appear to interact normally with *C*. *posadasii* endospores.

## Introduction

Fungal pathogens—often overshadowed by bacteria and viruses—now have become a greater global threat than ever before [1–3]. Recent reports on the rapidly growing impact of fungal diseases [4] underline the need for a better understanding of the mechanisms that govern the immune defense against fungi, in particular the innate response ranging from the initial recognition of fungal invaders to their neutralization.

**Fig 1:**
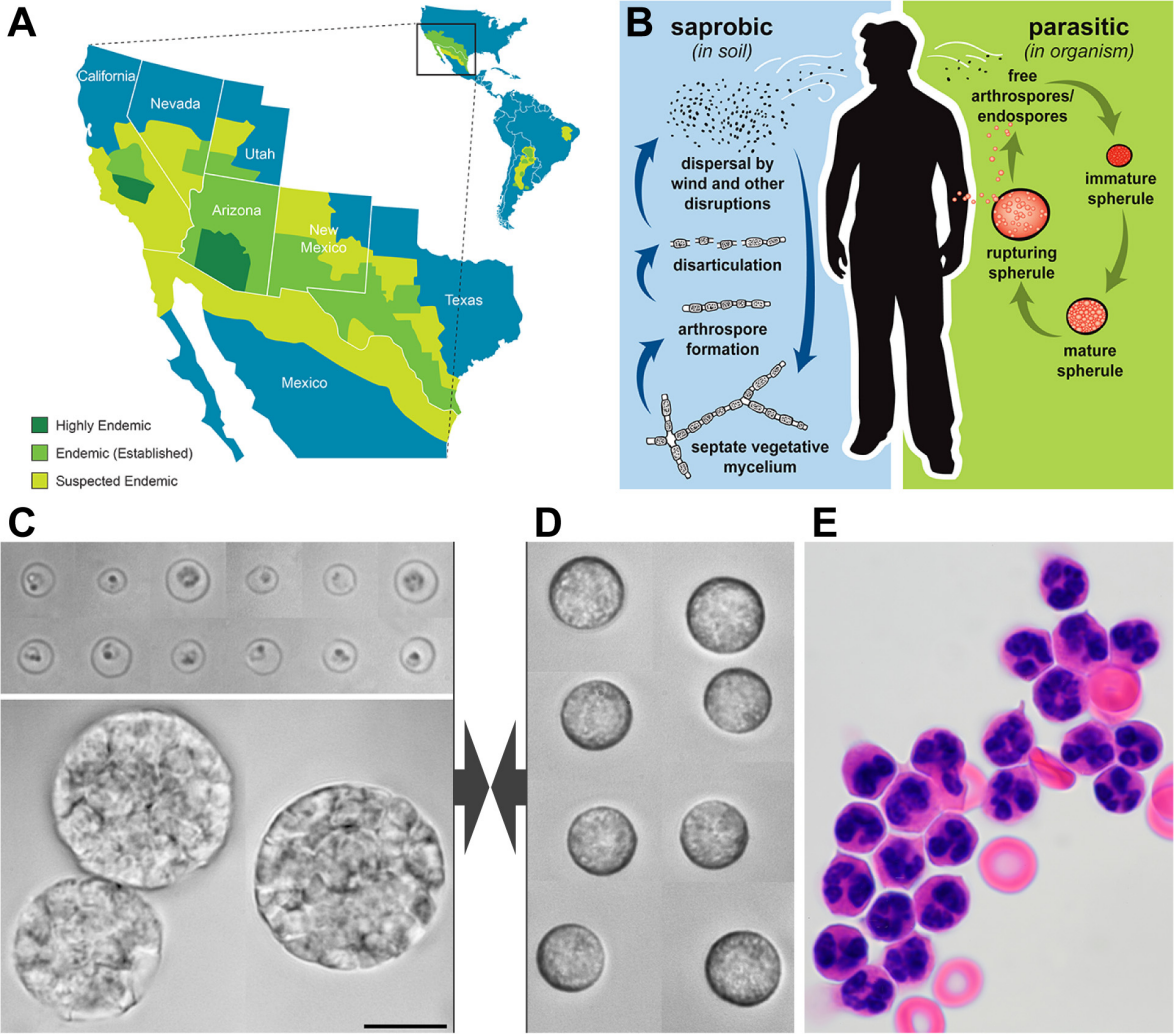
Overview of *Coccidioides* spp. and human neutrophils. A. Endemic areas of *Coccidioides* spp. B. Life cycle of *Coccidioides* spp. Vegetative mycelia exist in the soil and produce arthrospores during periods of low precipitation. Following aerosolization and inhalation of arthrospores, immature spherules develop and transition into large spherules containing hundreds of endospores. The mature spherules eventually rupture and release the endospores, reinitiating the spherule/endospore phase. (Adapted from [12] with permission.). C. Composite videomicrographs of typical *C*. *posadasii* endospores (*top*) and spherules (*bottom*). D. Composite brightfield videomicrographs of quiescent human neutrophils as used in the experiments. E. H&E-stained human neutrophils after neutrophil enrichment. All images in C-E are shown at the same magnification (some cell shrinkage occurred during H&E-staining). The common scale bar denotes 10 μm.

Coccidioidomycosis (commonly known as Valley fever) refers to the spectrum of disease caused by the fungi *Coccidioides immitis* and *Coccidioides posadasii* [5]. Coccidioidal infections primarily affect mammalian species in the Desert Southwest (Fig 1A) [6–8]. The incidence of coccidioidomycosis in humans continues to rise: a recent study estimated that the number of reported cases has increased 10-fold since 1998 [9]. Primary coccidioidal pneumonia accounts for 17–29% of all cases of community-acquired pneumonia in endemic regions [10–12]. Subsequent immunity to future infection is the norm; however, a minority of patients develop chronic infections, such as meningitis, requiring life-long antifungal therapy [13]. The high rate of infectivity mandates laboratory work with this pathogen be conducted in a biosafety-level-(BSL)-3 facility [14].

*Coccidioides* spp. are dimorphic fungi with a unique life cycle (Fig 1B). In the environment *Coccidioides* spp. exist primarily as a mold. During periods of low precipitation, septate hyphae undergo disarticulation, and aerosolized arthroconidia can be inhaled by animal hosts [15–17]. About 70–80% of invasive arthroconidia appear to survive the initial encounter with the host's immune system [18–25] and develop into immature spherules. The spherules (15–60 μm) mature and eventually burst, releasing hundreds of endospores (2–7 μm), which later grow to form new spherules, thus reinitiating the *in vivo* life cycle of this pathogen [26].

The unique pathogenesis, unusual resilience, and potential severity of coccidioidal infection highlight the need for dedicated studies of host interactions with *Coccidioides* spp. In fact, differences in the clinical manifestations of coccidioidomycosis, aspergillosis, candidiasis, and cryptococcosis, and dissimilarities between immune-cell interactions with different fungi [27], indicate that fungal recognition does not follow a single, universal route. Therefore, sound understanding of the mechanisms of fungal and other infections must be established one pathogen at a time. It requires the systematic dissection of the roles of each type of immune cell at various stages of the host defense, including immune-cell recruitment from a distance, close-up chemotactic distinction between the actual pathogen and cytokine-producing host cells, adhesive capture of pathogen particles, and their neutralization by phagocytosis. Furthermore, mounting evidence of poor correlation between animal models and human immune behavior [28–30] calls for increased efforts to study pathogen recognition by human immune cells.

To meet most of these challenges, we present a detailed look at the time courses of one-on-one interactions between human neutrophils and two distinct forms of *Coccidioides posadasii*. No clinical differences between *C*. *immitis* and *C*. *posadasii* have been reported to date; therefore, we expect our results to be representative of neutrophil encounters with both *Coccidioides* species. Neutrophils are the most abundant type of innate immune cell and often constitute the first line of defense against infections; however, relatively little is known about their response to *Coccidioides* spp. The behavior of neutrophils from human donors is particularly poorly understood, mainly because mature neutrophils cannot be genetically manipulated or cultured [31]. We here apply a recently developed, single-live-cell/single-target approach [32–34] to mimic, visualize, and analyze encounters of individual human neutrophils with *C*. *posadasii* endospores and spherules. Although these single-cell experiments are technically demanding, they enable us to discriminate cell-target from cell-substrate interactions and provide exceptional control over cell-target contacts. Moreover, they allow us to quantify the time-dependent behavior of live human immune cells, and to assess chemotaxis, adhesion, and phagocytosis separately on a per-cell basis. Finally, the analysis of similarities and differences between the responses of non-adherent, initially quiescent neutrophils to *C*. *posadasii* and other fungal and model particles places our results in the context of target-specific fungal recognition by human neutrophils, and enables us to assess to what extent previous results obtained with model targets carry over to neutrophil interactions with clinically important pathogens.

## Results

### Detailed view of one-on-one interactions between innate immune cells and *C*. *posadasii*

Although a wide range of animal hosts of *Coccidioides* spp. have been identified [35, 36], reported coccidioidal infections are largely limited to mammalian species. Here, we focus on innate interactions between human neutrophils and parasitic forms of *C*. *posadasii* (Fig 1C–1E). To ensure that all recognition experiments commence from a common baseline, we initially maintain the neutrophils in a passive state. We judge the quiescence of the cells by their spherical shape and lack of adhesion to the substrate, which in this case is the PEG-coated bottom coverslip of our microscope chamber (Fig 1D). The *C*. *posadasii* particles used in this study are endospores and spherules. As demonstrated in Fig 1C, these fungal targets have distinct sizes. Neutrophils have been shown to be able to phagocytose multiple particles of the size of the smaller endospores (2–7 μm in diameter), but they can only partially engulf targets larger than ~11 μm [32, 37], which includes typical sizes of *C*. *posadasii* spherules.

**Fig 2:**
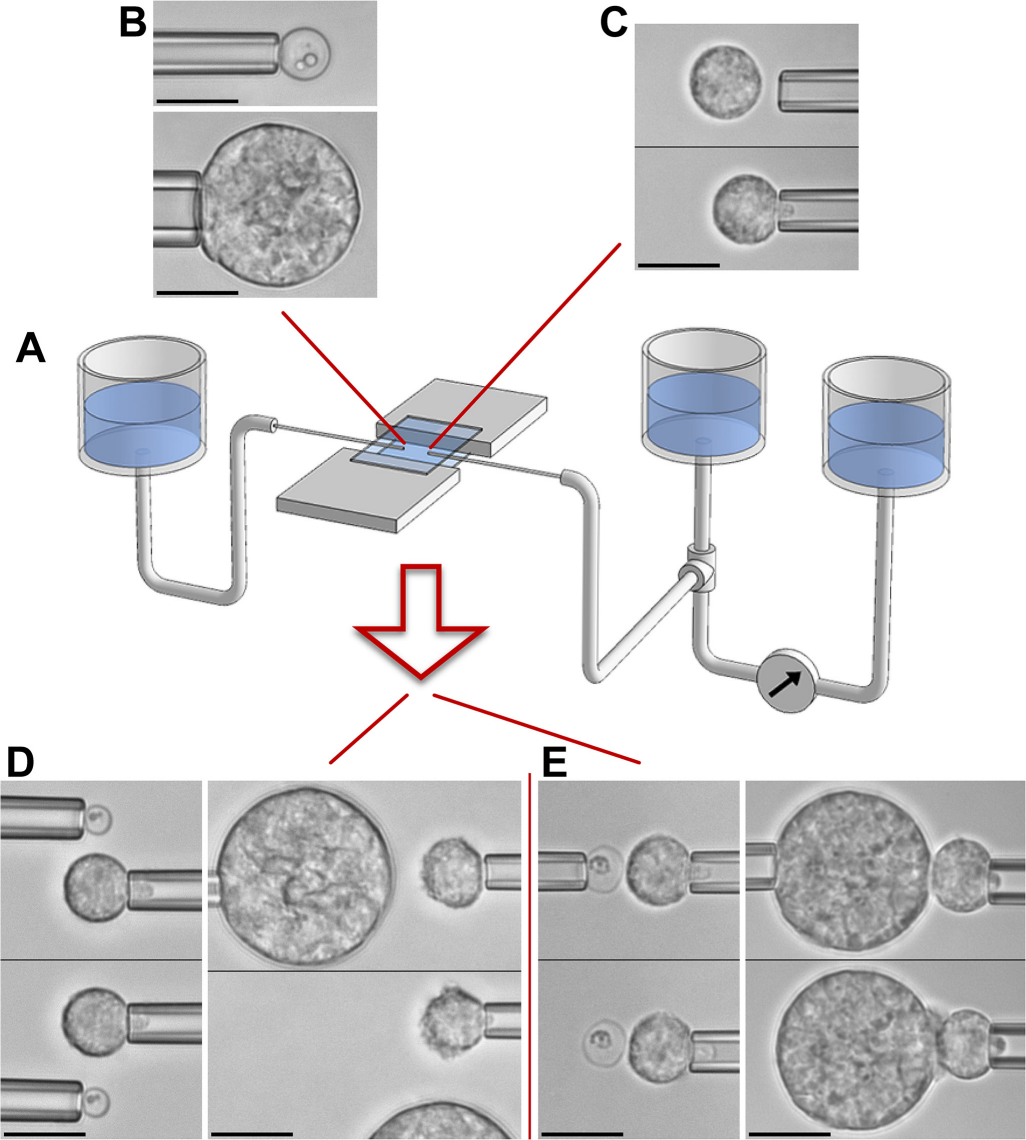
Overview of single-cell experiments. A. Schematic of our dual-micropipette manipulation system. The chamber volume is created by trapping buffer solution between two horizontal microscope coverslips. Facing pipettes access this volume through the chamber's two open sides. Vertically movable water reservoirs allow us to control the pipette-aspiration pressure with high resolution. The aspiration pressure of the right pipette is monitored in real time by measuring the height difference between the main reservoir (which is connected to the pipette) and a pre-zeroed reference reservoir. The included example videomicrographs demonstrate how micropipettes are used to pick up individual targets (B) and neutrophils (C) with gentle suction. After lifting these objects above the chamber bottom, they can be maneuvered in 3D to set up experiments that assess target recognition either from a distance (D) or upon direct physical contact (E). All scale bars denote 10 μm.

Fig 2 illustrates our *in vitro* approach to mimic encounters between a neutrophil (Fig 2C) and a chosen *C*. *posadasii* particle (Fig 2B) under well-controlled conditions. It is based on semi-automated dual-micropipette aspiration and manipulation (Fig 2A) [38]. Our experimental strategy integrates two stages that allow us to dissect vital immune-cell functions. The first stage (Fig 2D) addresses the question whether the cell is able to sense the target from a distance without direct physical contact. In the second stage, we maneuver the target into contact with the cell and then release it from its pipette (Fig 2E). Whether or not the cell keeps hold of the target provides a simple qualitative measure of the cell's aptitude to support cell-target adhesion. Finally, sustained cell-target contact usually initiates phagocytosis of the target within 1–3 minutes. Throughout this process, we record video images for later quantitative analysis of the time course of the single-cell/single-target interaction.

### *C*. *posadasii* induces complement-mediated chemotaxis of human neutrophils

The only way for an initially round cell to recognize a target over a distance of several micrometers is via chemical "messengers" emanating from the target. Visible changes of the cell morphology that correlate with the position of the target serve as confirmation that the cell has detected such chemicals. If a morphology change takes the form of a cellular protrusion directed toward the target, it is an indicator of chemotactic cell behavior. If non-adherent (e.g., pipette-held) cells exhibit such behavior, we call their response "pure chemotaxis", because it is unbiased by intracellular processes that otherwise simultaneously coordinate cell-substrate adhesion [33, 34, 39]. It is worth noting that our single-cell technique appears to be the only chemotaxis assay currently in existence that is able to distinguish chemotactic sensing from the interfering effects of cell-substrate adhesion.

**Fig 3:**
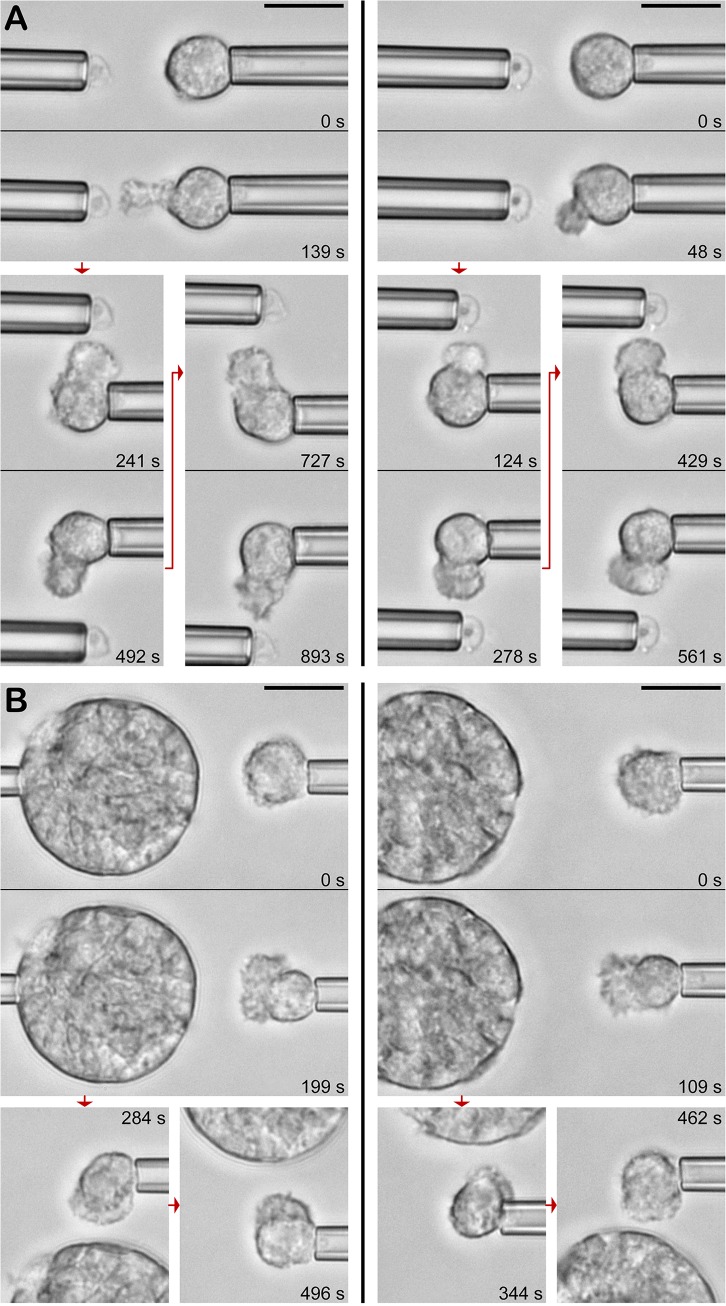
Pure chemotaxis of non-adherent neutrophils toward *C*. *posadasii*. Using micropipettes, endospores (A) (see also S1 Video) and spherules (B) are maneuvered to different positions relative to the cell without touching the cell. In this configuration, chemotaxis takes the form of a directional, protrusive pseudopod extended by the neutrophil toward the target. The relative times of all video images are included. Table 1 summarizes the number of experiments in which this behavior was observed. All scale bars denote 10 μm.

Our single-cell experiments revealed that quiescent neutrophils readily recognize individual *C*. *posadasii* particles without physical contact (Fig 3, S1 Video). In the presence of 10% autologous serum, pipette-held neutrophils detected both endospores as well as spherules of *C*. *posadasii* from a distance of 5–15 μm (Table 1). This recognition distance generally appeared to be larger for spherules than for endospores. Each cell typically responded to the nearby *C*. *posadasii* particle by extending a directional pseudopod toward the particle (Fig 3). When the particle was moved away, the cell retracted this purely chemotactic pseudopod. Likewise, when the particle was relocated to a different side of the cell, the cell retracted the previous pseudopod within 1–2 minutes while extending a fresh pseudopod toward the new target position (see S1 Video for 3 examples of neutrophil-endospore pairs). We did not observe this pure chemotaxis in serum-free buffer or in buffer containing heat-treated serum and, therefore, attribute it to the production of chemoattractant anaphylatoxins by the complement system at the surface of the fungal particles.

**Table 1:** Recognition of endospores and spherules of *Coccidioides posadasii* by human neutrophils.

	Endospores		Spherules	
	10% normal autologous serum	10% heat-treated autologous serum	10% normal autologous serum	10% heat-treated autologous serum
**Chemotaxis**	7 (9)	0 (21)	10 (10)	0 (8)
**Adhesion**	24 (26)	22 (25)	34 (34)	7 (7)
**Phagocytosis**	23 (26)	18 (22)	34 (34)	7 (7)

The table lists the numbers of single-cell experiments in which clear chemotactic, adhesive, and phagocytic interactions between neutrophils and *Coccidioides* particles were observed. The number in parentheses is the total number of experiments testing for the respective type of interaction.

Interestingly, the cells were not only able to locate the direction from which the chemoattractant emanated, but also tended to form pseudopods whose shapes correlated, to some extent, with the size of the *C*. *posadasii* particles. We assessed the morphological difference between pseudopods protruding toward (small) endospores (Fig 3A) and (large) spherules (Fig 3B) by measuring the size of the pseudopodial base as a fraction of the original cell-surface area. This difference was most distinctive during the initial cell deformation, i.e., after a target particle was first brought into the vicinity of the cell. During the chemotactic response to endospores, the pseudopodial base extended over ~18±11(SD)% of the original neutrophil surface (*N* = 7 pseudopods). In contrast, *C*. *posadasii* spherules tended to induce the formation of chemotactic pseudopods with broader bases, perturbing ~38±13(SD)% of the original cell surface (*N* = 12 pseudopods). The difference was statistically significant (with a *p*-value <0.003). This observation might hold important clues about currently poorly understood cellular mechanisms governing the directional cell response to chemotactic stimuli [40].

### Human neutrophils readily adhere to and phagocytose *C*. *posadasii* endospores and spherules

Once a cell was pre-activated by chemotactic stimulation, subsequent physical contact with a *C*. *posadasii* particle usually resulted in cell-target adhesion, followed by the swift phagocytic uptake of the particle (Table 1). However, this type of response entailed elements of both continued chemotaxis as well as phagocytosis [33]. To assess instead pure phagocytosis of *C*. *posadasii* by initially passive neutrophils, we also performed a set of experiments in buffer containing heat-treated autologous serum [34]. Heat-treated serum acts to passivate the glass surfaces of the coverslip and the pipette, helping to prevent premature activation of the cells [41]. Under these conditions, neutrophil chemotaxis was completely suppressed (as tested with *N* = 21 endospores and *N* = 8 spherules; Table 1). The same conditions also had been used in experiments with other targets such as zymosan particles [34] and, therefore, allowed us to directly compare the neutrophil behavior in encounters with *C*. *posadasii* and those targets.

**Fig 4:**
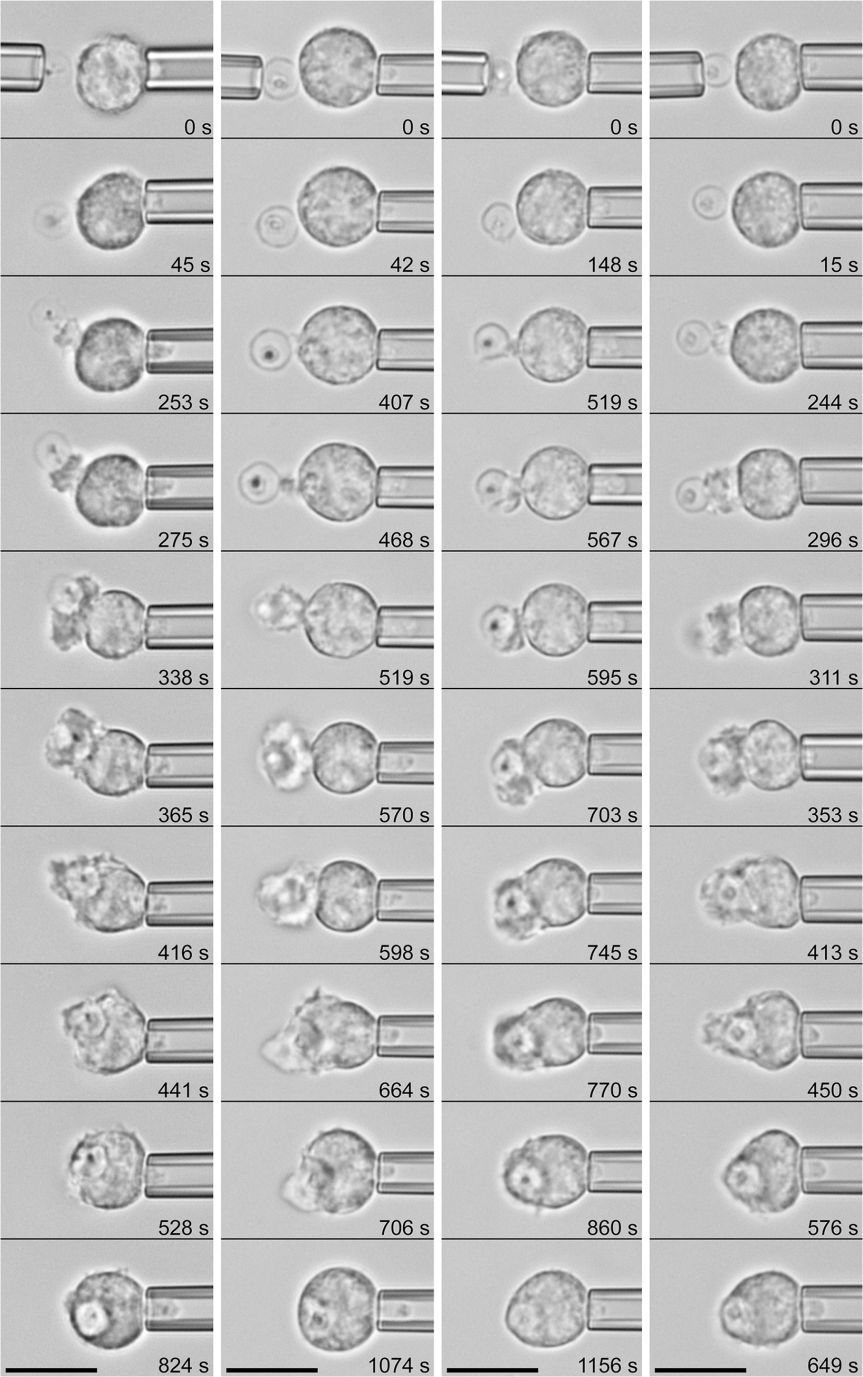
Phagocytosis of *C*. *posadasii* endospores by initially passive human neutrophils. (See also S2 Video.) Four example experiments are presented as vertical filmstrips. The experiment buffer contained 10% heat-treated autologous serum, which prevented chemotaxis (such as shown in Fig 3) but not the engulfment of the endospore after direct contact with the neutrophil surface. The relative times of all video images are included. Table 1 summarizes the number of experiments in which this behavior was observed. All scale bars denote 10 μm.

**Fig 5:**
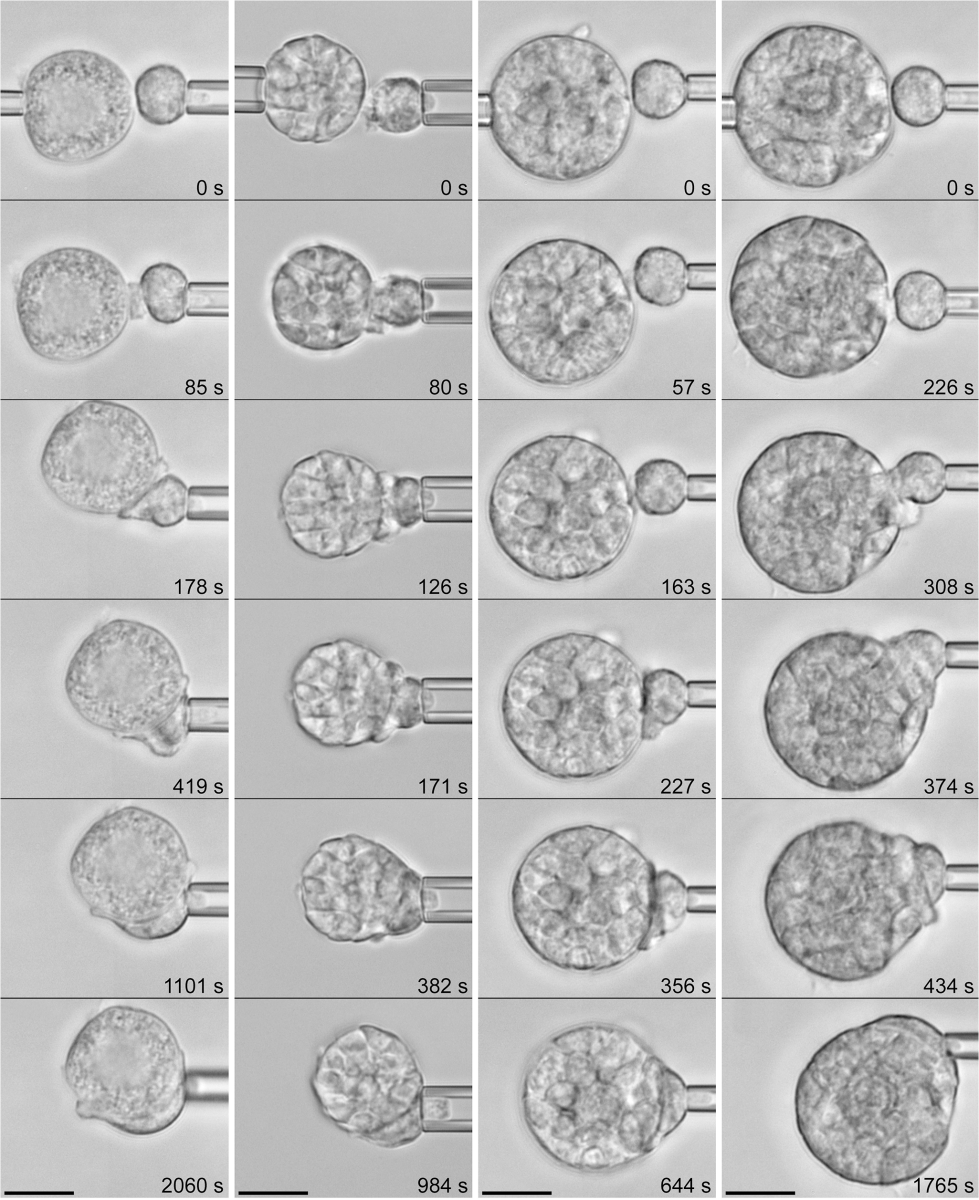
Phagocytosis of *C*. *posadasii* spherules by initially passive human neutrophils. (See also S3 Video and S4 Video.) Four example experiments are presented as vertical filmstrips. (The spherule in the first panel is immature [71].) The experimental conditions were the same as in Fig 4. The relative times of all video images are included. Table 1 summarizes the number of experiments in which this behavior was observed. All scale bars denote 10 μm.

Examples of typical phagocytosis experiments with *C*. *posadasii* endospores and spherules in buffer containing 10% heat-treated serum are shown in Figs 4 and 5, respectively (see also S2–S4 Video). We found that human neutrophils generally responded strongly to direct physical contact with either of these two target types even when the supplemented serum had been heat-treated (Table 1). In contrast, in control experiments in the absence of serum (data not shown), we observed some adhesion between neutrophils and *C*. *posadasii* particles, but never chemotaxis nor phagocytosis. Together, the neutrophil behavior under these two conditions indicated that some components of heat-treated serum still opsonized the fungal surfaces, in agreement with previous results obtained with zymosan [34].

The neutrophils readily engulfed individual endospores. In this case, phagocytosis commenced with a protrusive cell deformation local to the attached endospore, leading to the formation of a pseudopodial "pedestal" that at first pushed the target outwards (i.e., away from the main cell body, as seen in images 3–5 of all four filmstrip examples of Fig 4). Within 2–3 minutes, the growing chemotactic-like pseudopod appeared to overflow and completely surround the endospore. Eventually, the cell resumed a more or less spherical shape, and in the process of rounding up, it "pulled" the endospore inwards (see S2 Video for 3 examples).

As expected, single neutrophils could not completely surround the large spherules of *C*. *posadasii*, exhibiting frustrated phagocytosis instead (Fig 5). Nevertheless, in contrast to an earlier report [22], the neutrophil response to spherules was vigorous (Table 1) and appeared to be independent of the maturation state of the spherules (shown in S3 Video for an immature spherule and in S4 Video for a mature spherule). We note that because of the greater weight of the spherules, we had to increase the duration of the initial contact between each pair of cell and spherule before releasing the latter from its holding pipette. The resulting firmer cell-target attachment helped to prevent the spherule from quickly sinking out of focus; however, the longer enforced contact tended to obscure the early cell response somewhat. Nonetheless, we again observed that phagocytosis by most neutrophils commenced with the formation of a characteristic protrusive pedestal, similar to the cell behavior at the onset of the engulfment of endospores.

**Fig 6:**
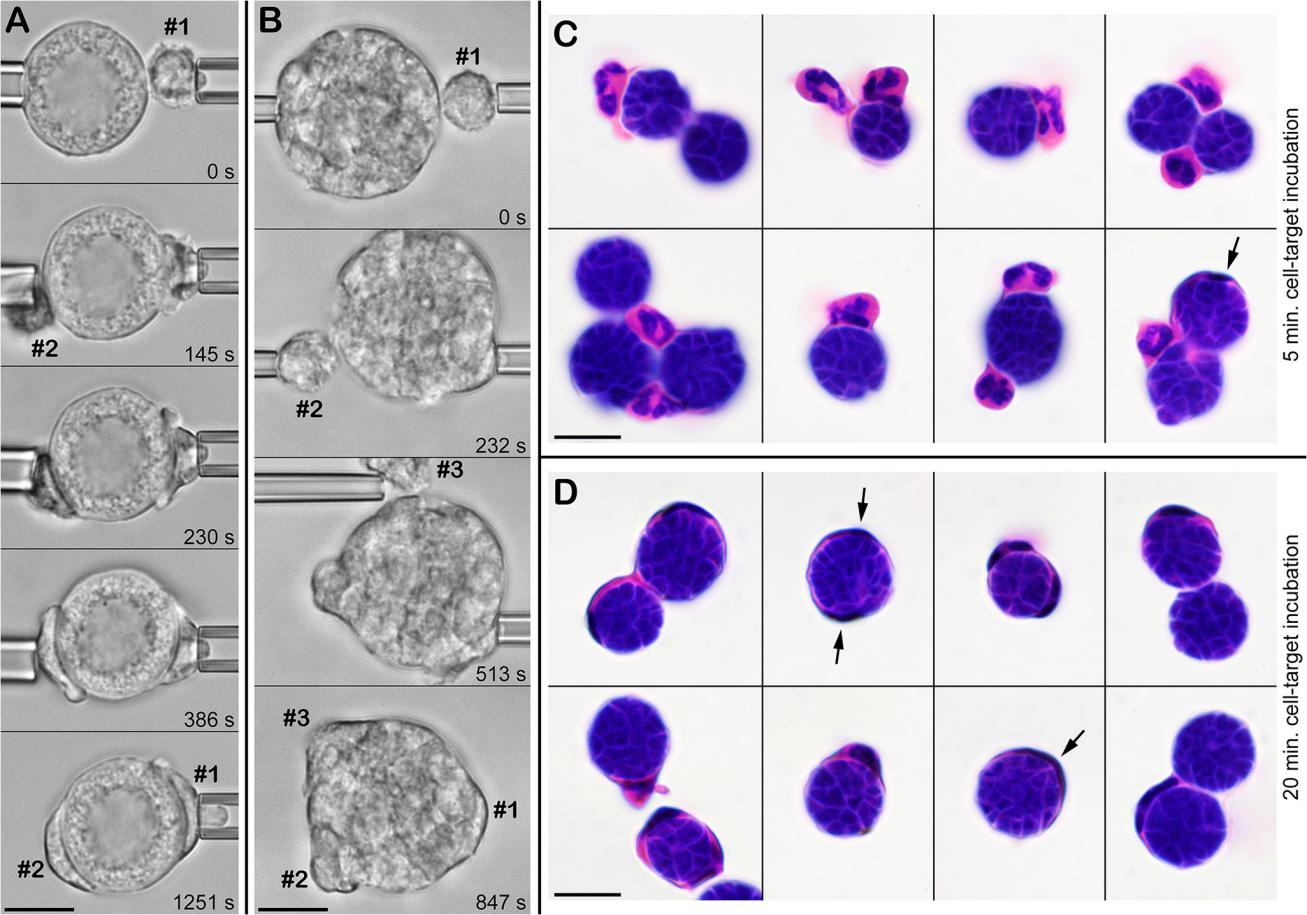
Frustrated phagocytosis of *C*. *posadasii* spherules by multiple neutrophils. A. Shortly after manipulating an immature spherule and a first neutrophil (#1) into contact, a second neutrophil (#2) touches and adheres to the spherule by chance. Both cells proceed to spread over the surface of the spherule. (See also S5 Video.) B. Using micropipettes, three neutrophils are sequentially brought into contact with the same spherule and proceed to attack it. (See also S6 Video.) The relative times of all video images are included. C,D. Bulk assay to verify the recognition of live *C*. *posadasii* spherules by human neutrophils. The spherules were incubated with neutrophils for 5 min. (C) or for 20 min. (D) in suspension with gentle mixing on a rotator, then fixed and H&E-stained. Arrows point to particularly spread-out leukocytes. (Some cell shrinkage occurred during H&E-staining.) All scale bars denote 10 μm.

As long as part of the spherule surface remained uncovered by adherent neutrophils, contact with additional cells (either by chance, as in Fig 6A, or as a result of micropipette manipulation, as in Fig 6B) led to strong adhesion as well, and resulted in frustrated phagocytosis of individual spherules by multiple neutrophils (shown in S5 Video for an immature spherule and in S6 Video for a mature spherule).

To verify that the strong neutrophil response to contact with *C*. *posadasii* particles was not a consequence of our thimerosal treatment to inactivate the fungus, we also performed recognition assays with untreated live *C*. *posadasii* particles. Unfortunately, we are unable to set up our dual-micropipette system in a BSL-3 laboratory and, therefore, cannot carry out single-cell experiments with live *C*. *posadasii* particles. We instead assessed neutrophil interactions with untreated live *C*. *posadasii* in bulk, by incubating cells and *C*. *posadasii* particles (in the presence of autologous serum) in suspension on a rotator for various amounts of time. Remarkably, even when the cell-target incubation time was as short as 5 minutes, there was ample evidence of a vigorous adhesive and phagocytic response of neutrophils to live spherules (Fig 6C) and endospores (not shown). After 20-minute incubation (Fig 6D), many neutrophils were spread so thinly over spherules that their reliable identification required staining (here: using H&E stain to visualize the spread-out neutrophils).

### Quantitative analysis of the phagocytic uptake of *C*. *posadasii* endospores and spherules

**Fig 7:**
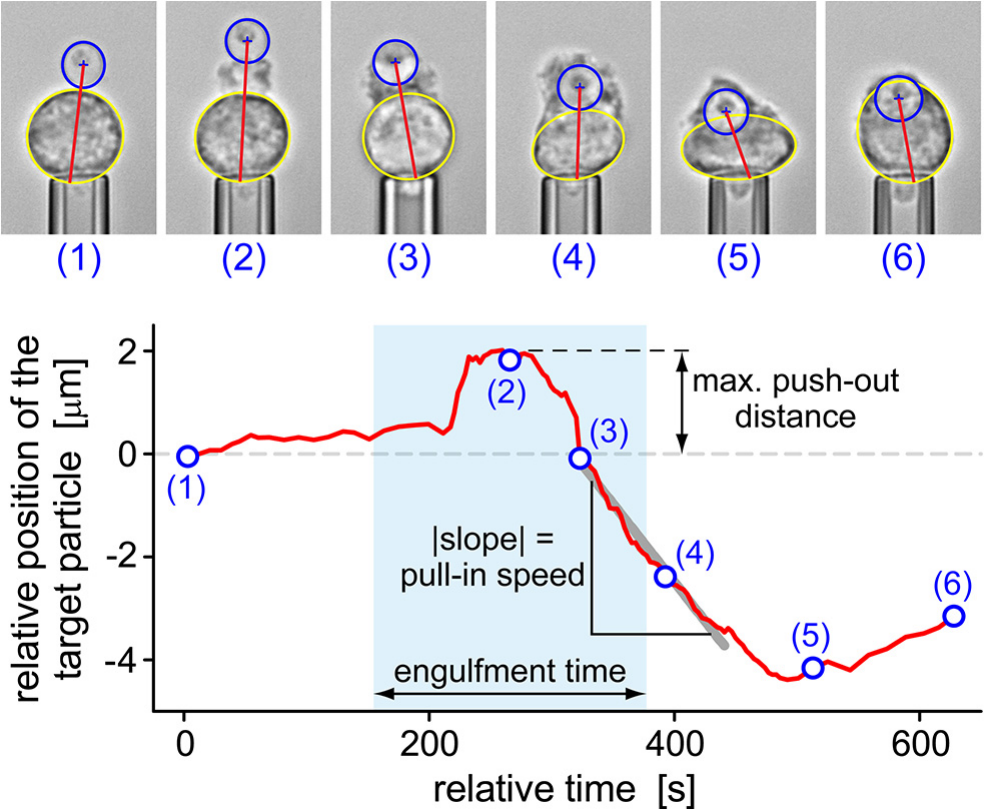
Analysis of the positional trajectory of a target particle (here: a *C*. *posadasii* endospore) during phagocytosis by a pipette-held neutrophil. The annotated videomicrographs at the top demonstrate our measurement of the distance between the center of the target particle and the opposite side of the main cell body (*red straight line*). This distance (relative to its initial value) is plotted as a function of time in the bottom graph (*red curve*). Numbered circles correspond to the time points at which the respective example images were taken. This type of graph allowed us to determine the maximum push-out distance as well as the pull-in speed of the target as shown. The engulfment time (defined in the text) was found by inspection of the recorded video images.

Our single-cell/single-target approach allowed us to quantify the time course of phagocytosis by inspecting the positional trajectory of the target as it is taken up by the cell, the cell-surface area, the cortical tension (i.e., the mechanical resistance of the cell to expansion of its apparent surface area [32]), and the total engulfment time. Fig 7 illustrates the analysis of the time-dependent trajectory of a *C*. *posadasii* endospore during phagocytosis. In addition to exposing detailed neutrophil behavior throughout target engulfment, this analysis furnished the values of parameters such as the maximum push-out distance and the pull-in speed of the target as defined in Fig 7. Furthermore, we defined the engulfment time as the period from the first unambiguous sign of visible cell deformation local to the attached target to the closure of the phagocytic cup. Statistical analyses of these three parameters are included in a later section.

**Fig 8:**
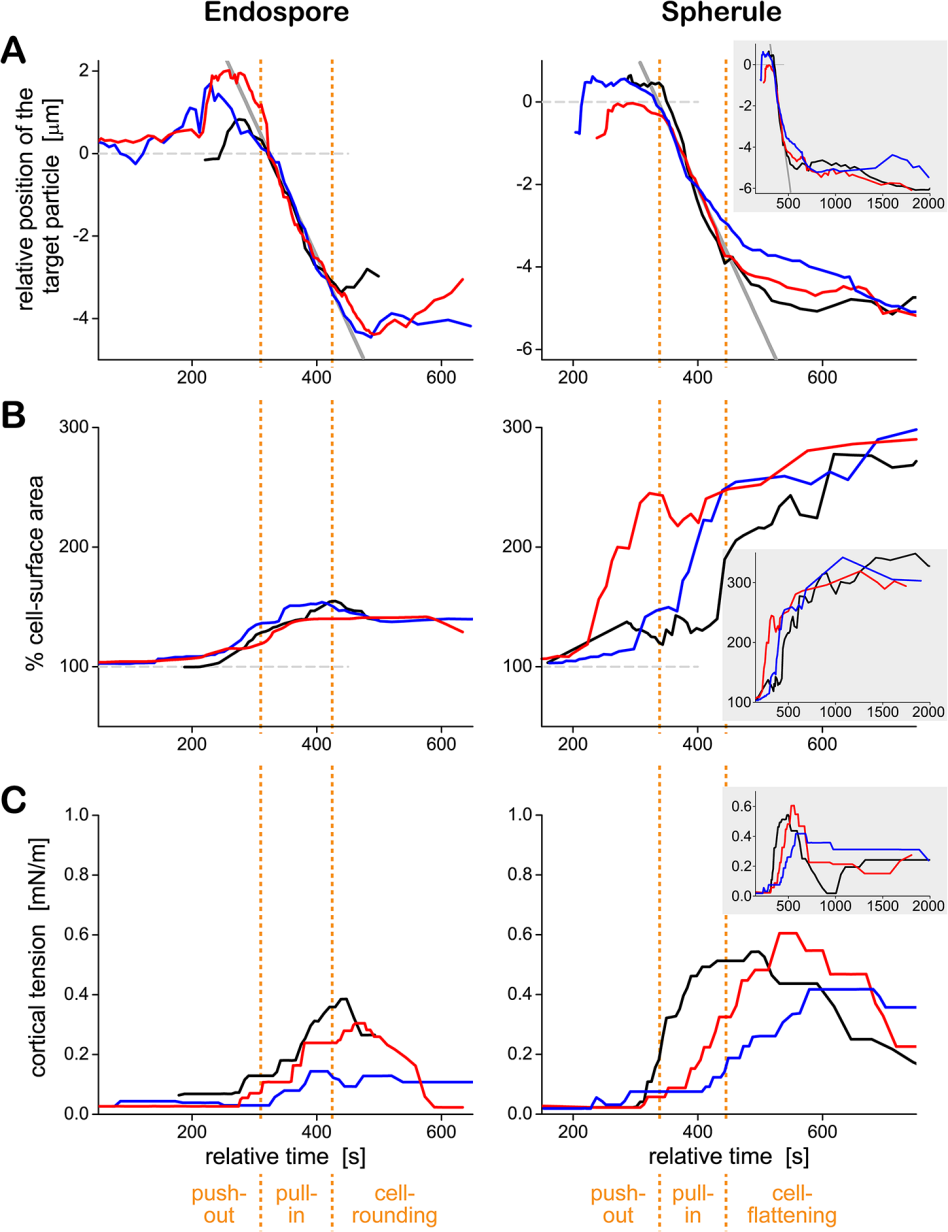
Quantitative analysis of the time course of phagocytosis of *C*. *posadasii* endospores (*left*) and spherules (*right*) by human neutrophils. Representative timelines of the target position (aligned for best overlap of the trajectories during the pull-in phase) (A), the cell-surface area (B), and the cortical tension (C) are shown for three particles of each type. For each target type, a given color indicates the same cell-target pair throughout parts A, B, and C. The three phases identified at the bottom of the figure were determined by inspection of the time-dependent neutrophil morphologies in the recorded image sequences. Positive values of the target position shown in part A reflect a push-out of the particle. A monotonous decrease of the position values characterizes the pull-in phase. The end of the pull-in phase marks the start of the final phase. The inset in the right panels depicts the cell behavior over an extended period of time (~33 minutes). Common axis titles are shown only once at the left and bottom of the figure.

Fig 8A presents typical target trajectories of *C*. *posadasii* endospores and spherules side by side, along with the respective timelines of the cell-surface area (Fig 8B) and the cortical tension (Fig 8C). Three examples were selected each from *N* = 18 and *N* = 7 single-cell experiments with endospores and spherules, respectively. They illustrate the typical neutrophil behavior and typical variability of the measured timelines during cell-target interactions in the presence of heat-treated serum. Three distinct phases of the phagocytosis of *C*. *posadasii* particles are identified at the bottom of the figure. The first two—a target push-out phase, followed by a fairly rapid target pull-in phase—were common to both endospores and spherules. The third phase was target-size dependent. In the case of (now fully engulfed) endospores, this was a cell-rounding phase. The neutrophil resumed a more or less spherical shape characteristic for a passive cell. In the case of the frustrated phagocytosis of spherules, the last phase was a cell-flattening phase during which the neutrophil spread over as much target surface as possible.

The data of Fig 8A confirmed that contact between a neutrophil and either type of *C*. *posadasii* particle first triggered the formation of a chemotactic-like cell protrusion that displaced the particle outwards by a small distance. About 1–2 minutes later, the direction of target motion reversed, signifying that the cell started to “pull in” the *C*. *posadasii* particle. The rates of displacement of both endospores and spherules were similar during this phase (as indicated by the gray solid lines, which have the same slope). Intriguingly, the start of this pull-in phase often coincided with the onset of the increase of the cortical tension (Fig 8C). This concurrence suggests that the primary mechanism of the inward motion of target particles is based on the cortical tension (rather than molecular motors that might directly pull or push the particles toward the cell center) [42, 43].

In most experiments, the apparent cell-surface area (Fig 8B) started to increase earlier than the cortical tension (Fig 8C), confirming that the plasma membrane of passive neutrophils has a certain amount of “slack” [32]. In other words, neutrophils can accommodate small deformations (involving surface-area increases of up to ~30% [32]) without having to cope with a significantly elevated mechanical resistance that otherwise accompanies (larger) increases of their surface area.

Concluding the mechanical processes during the phagocytosis of endospores, the cell-rounding phase was characterized by the relaxation of the surface area to a new minimum. The total final area consisted of two parts: the area of the membrane surrounding the internalized particle (e.g., the surface of the phagosome), and the area of the outer cell envelope that now enclosed the combined volume of cytoplasm and particle. As expected, this final area correlated with the size of the engulfed endospores; it typically was ~40% higher than the initial surface area of the resting neutrophil. In contrast, there was no consistent reduction in surface area in the cell-flattening phase during frustrated phagocytosis of *C*. *posadasii* spherules. Instead, the surface area of neutrophils appeared to gradually approach a plateau at ~300% of the area of resting cells. This value agrees with previous estimates of the maximum apparent surface area that neutrophils can generate during phagocytosis [37, 41]. Finally, the maximum cortical tension measured during frustrated phagocytosis of spherules was significantly higher than during the uptake of endospores (Fig 8C), in agreement with a previous assessment of the surface-area/tension relationship of human neutrophils during phagocytosis of particles of different sizes [37].

### Comparison with other fungi marks the close-range response to *C*. *posadasii* as one of the strongest antifungal responses by human neutrophils

The response of innate immune cells to microbes can vary dramatically depending on the type and state of host cells as well as the mixture of native constituents and opsonins displayed on the pathogen surface [27, 41, 43]. Unfortunately, despite a large body of existing work on immune-cell interactions with various fungi, the lack of standardized experimental approaches prohibits direct quantitative comparisons of literature reports. For example, neutrophils in bulk assays are often stimulated in various ways, e.g., through cell-substrate adhesion (which can increase the production of reactive oxygen intermediates as much as 100fold under otherwise identical conditions [44]), by addition of priming agents, or simply because a fraction of the short-lived cells undergo apoptosis. As a result, such studies tend to mimic an inflammatory-like setting rather than address the question if, and how well, initially quiescent neutrophils recognize a particular type of microbe.

In ongoing work we are using our highly discriminatory single-cell approach to compare immune-cell responses to various types of microbe. Here, we provide a summary of our findings on the human neutrophil responses to seven different targets, all assessed under identical conditions. In addition to *C*. *posadasii* particles, the targets are *Candida albicans*, *Cryptococcus neoformans*, *Aspergillus fumigatus*, *Rhizopus oryzae*, zymosan particles, and antibody-coated microspheres. Zymosan is a particulate, insoluble fraction from yeast cell walls that consists mostly of the polysaccharides glucan and mannan (~75% of the dry mass) [45–47] and is often used in *in vitro* studies of chemotaxis and phagocytosis. The antibody-coated polystyrene beads used here were prepared in such a way that their surfaces primarily exposed Fc domains of immunoglobulin G (IgG; cf. Materials and Methods). Except for *C*. *posadasii* spherules, most targets had roughly the same size (3–5 μm in diameter), although *C*. *albicans* and *C*. *neoformans* cells tended to be slightly larger (5–6 μm), and *A*. *fumigatus* conidia slightly smaller (2.5–3.5 μm), than the overall average size. The primary purpose of this comparison was to evaluate how the response to *C*. *posadasii* compares to neutrophil interactions with other common pathogenic fungi, and thus to place the results of the previous sections into a broader context. While details of our work with the other fungal targets are beyond the scope of this paper, we note that a direct comparison of immune-cell interactions with these seven targets is presented here for the first time.

**Fig 9:**
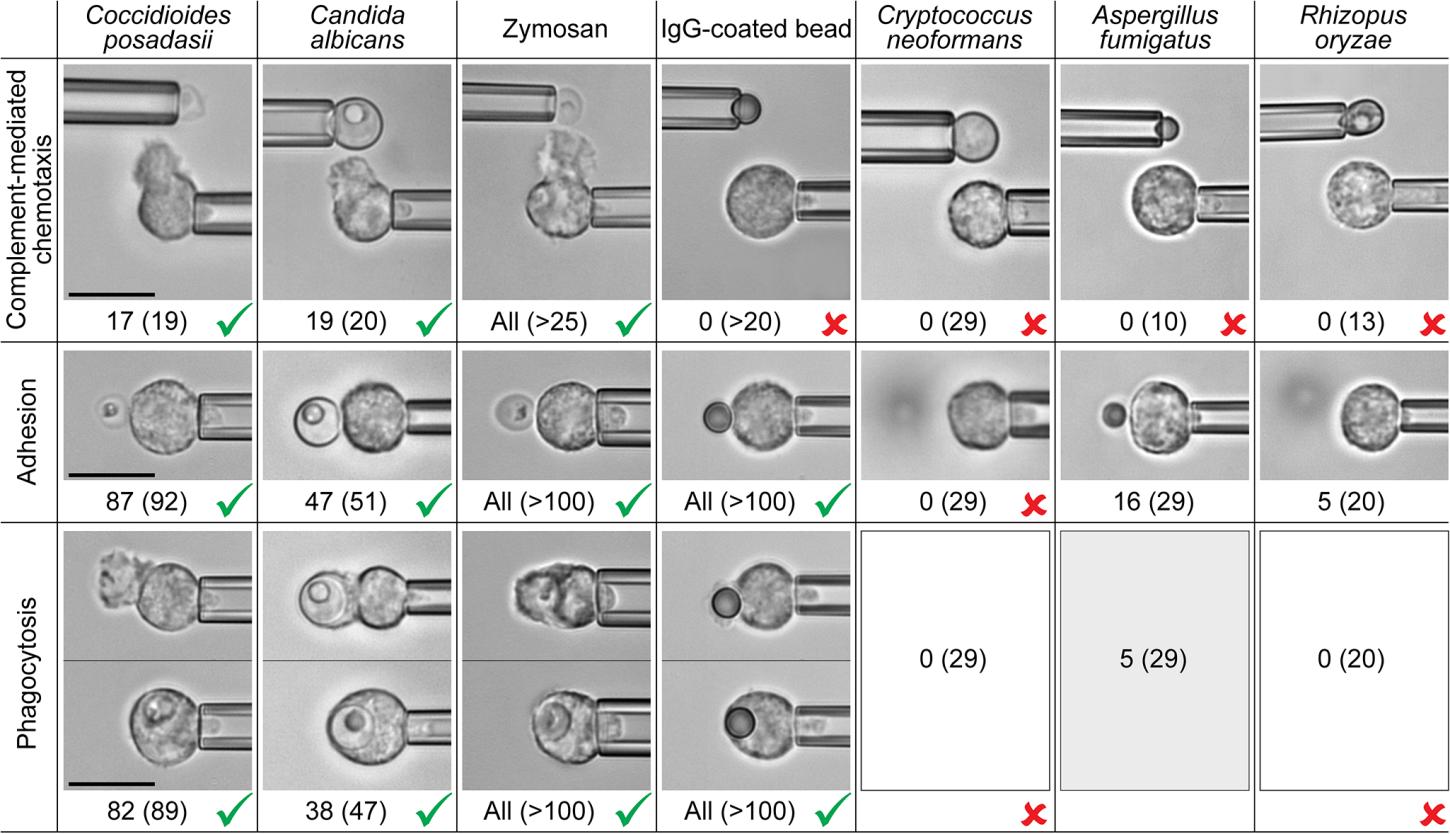
Side-by-side comparison of the aptitude of passive human neutrophils to recognize various fungal and model targets. Example videomicrographs show typical outcomes of single-cell experiments assessing chemotaxis (in buffer containing 10% autologous serum) as well as adhesion and phagocytosis (where in the experiments with *C*. *posadasii*, *C*. *albicans*, and zymosan, the buffer contained 10% of either normal or 52°-C-treated autologous serum). The number of positive responses and the total number of inspected cell-target contacts (in parentheses) are included. In the case of *C*. *posadasii*, the numbers comprise the cell responses to both endospores as well as spherules. The outcomes of experiments with zymosan and IgG-coated beads agree with, and include, results that were reported previously [34, 37, 43]. Unambiguous positive (>80% positive response) and negative (never observed) responses are marked by checkmarks and crosses, respectively. All scale bars denote 10 μm.

Fig 9 summarizes the aptitude of passive human neutrophils to recognize the chosen fungal and model targets, assessing separately chemotaxis, adhesion, and phagocytosis. Testing for pure chemotaxis of neutrophils, we found that *C*. *albicans* and zymosan particles elicited the same positive response as *C*. *posadasii* particles (Fig 9, cf. also Fig 3). All of these fungal surfaces triggered the formation of directional pseudopods by nearby neutrophils in buffer containing 10% normal, autologous serum. This chemotactic response was suppressed in the absence of serum, or when we used serum that had been heat-treated (at 52°C or higher). In contrast, we did not observe complement-mediated neutrophil chemotaxis toward *C*. *neoformans*, *A*. *fumigatus*, and *R*. *oryzae*, or toward antibody-coated beads (Fig 9).

Inspecting the adhesive response of neutrophils to direct contact with the chosen targets under conditions that suppressed chemotaxis (i.e., in the presence of heat-treated serum), we found that the cells adhered strongly to *C*. *posadasii*, *C*. *albicans*, zymosan, and antibody-coated beads. In contrast, quiescent neutrophils were unable to adhere to the surface of the polysaccharide capsule of *Cryptococcus neoformans* [48, 49], in agreement with previous reports [50, 51], and unlike neutrophils that were activated in various ways [52]. Despite enforced firm and prolonged cell-target contact, only 16 out of 29 neutrophil contacts with *A*. *fumigatus*, and 5 out of 20 contacts with *R*. *oryzae*, resulted in sustained adhesion. Compared to the strong adhesive interactions of neutrophils with *C*. *posadasii*, *C*. *albicans*, and zymosan, the level of adhesion to *A*. *fumigatus* was weak to moderate, whereas adhesion to *R*. *oryzae* was very weak (Fig 9).

Without cell-target adhesion, phagocytosis is impossible, as reflected in Fig 9 for *C*. *neoformans*. Other than that, we found that initially quiescent human neutrophils readily phagocytosed antibody-coated beads independent of the presence of serum. Phagocytosis of *C*. *posadasii*, *C*. *albicans*, and zymosan particles proceeded vigorously provided these fungal particles were opsonized with serum (even in cases where the serum had been heat-treated at 52°C, which are included in Fig 9). In the absence of serum, this phagocytosis was suppressed. In contrast, only a few contacts of neutrophils with *A*. *fumigatus* conidia (5 out of a total of 29, in which case all 5 neutrophils were from the same donor) resulted in successful phagocytosis. Our observation that initially quiescent neutrophils exhibit moderate adhesion to *A*. *fumigatus* conidia, but fail to phagocytose most of them, appears to be consistent with previous studies [27, 53, 54]. We did not observe any phagocytosis of *R*. *oryzae* particles independent of their stage of germination (Fig 9).

In those cases where target particles triggered a strong phagocytic response, we analyzed the time course of phagocytosis as explained in the previous section. The most striking outcome was a clear difference between the neutrophil response to antibody-coated beads on the one hand, and to the fungal particles (*C*. *posadasii* endospores, *C*. *albicans* cells, and zymosan particles) on the other. Whereas the initial stage of the engulfment of *C*. *posadasii*, *C*. *albicans*, and zymosan particles typically involved the formation of a protrusive pedestal (cf. Fig 4), the cell morphology during phagocytosis of antibody-coated beads generally lacked this distinctive protrusive deformation, in agreement with [43]. Instead, the beads were taken up in a more straightforward manner, starting with the formation of a comparatively thin phagocytic cup that advanced along the bead surface without displacing the bead outwards.

In summary, *C*. *posadasii* elicited one of the strongest responses during close encounters with human neutrophils. This response was similar in strength to neutrophil interactions with *C*. *albicans* cells and zymosan particles, but it clearly outperformed the neutrophil responses to *C*. *neoformans*, *A*. *fumigatus*, and *R*. *oryzae*. This surprising outcome contrasts with the currently accepted paradigm of the importance of neutrophils for protection against aspergillosis and mucormycosis. Furthermore, the similarities between neutrophil interactions with *C*. *posadasii* endospores and with zymosan particles encompassed not only the strengths of chemotaxis, adhesion, and phagocytosis, but also extended to the time-dependent neutrophil morphology during the engulfment of these targets. The fact that our discriminatory assays were unable to distinguish the neutrophil responses to these two target types, while revealing clear differences to cell interactions with antibody-coated beads, leads us to speculate that human neutrophils recognize similar immunogenic features on the surfaces of zymosan and *C*. *posadasii*.

### Comparison of neutrophils from healthy donors and patients with chronic coccidioidomycosis

**Fig 10:**
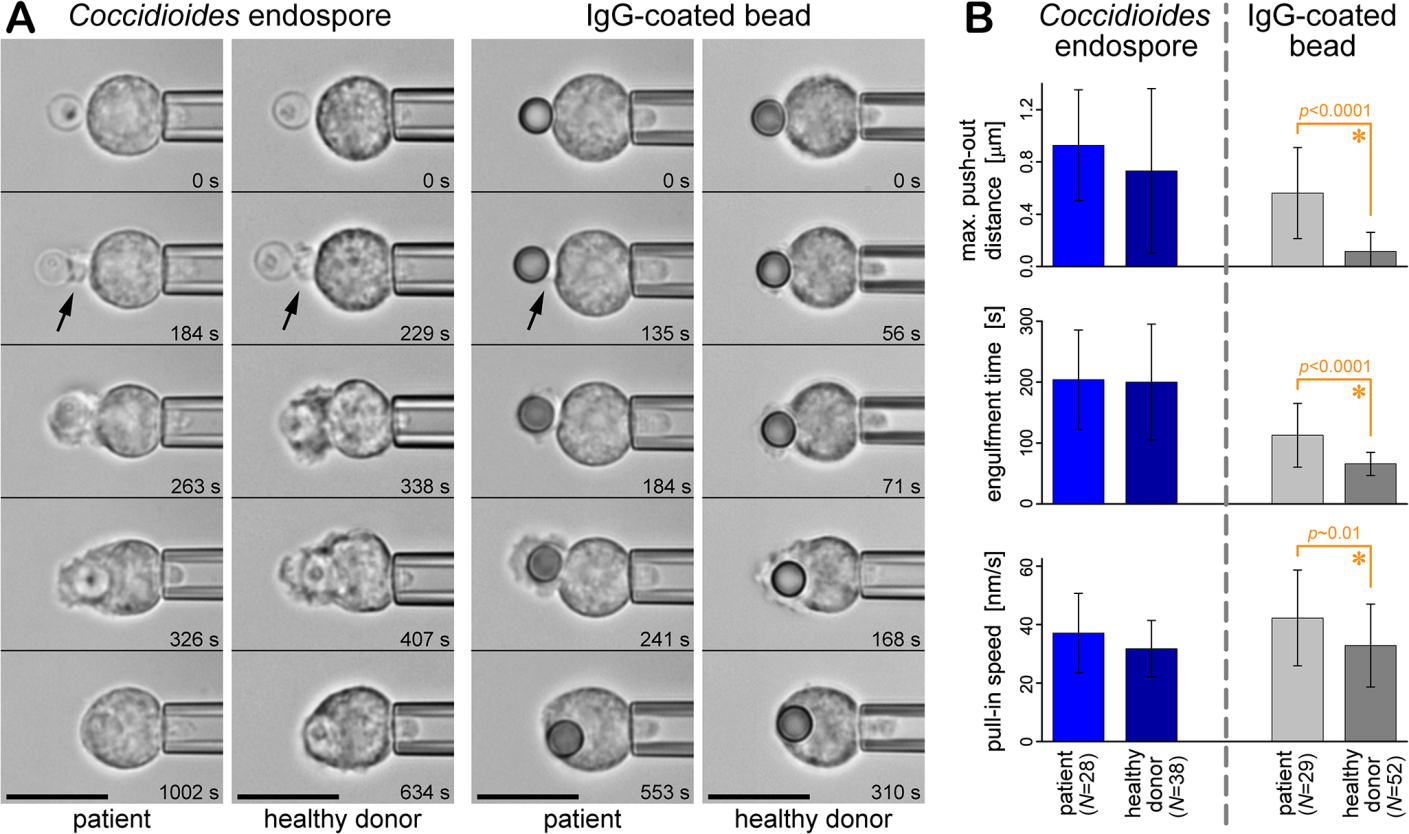
Comparison of the behavior of neutrophils from patients with chronic coccidioidomycosis and from healthy donors. A. Filmstrips illustrate the responses of neutrophils from the two donor groups to contact with *C*. *posadasii* endospores and antibody-coated beads, respectively (in the presence of autologous serum). All scale bars denote 10 μm. B. Results of the quantitative analysis of the positional trajectories (cf. Fig 7) of *C*. *posadasii* endospores and antibody-coated beads during phagocytosis by neutrophils from the two donor groups. (Error bars denote standard deviations, and asterisks mark differences that are statistically significant. The number *N* of analyzed single-cell experiments is included in the figure.).

In addition to neutrophils from healthy donors, we also examined neutrophils from eight patients suffering from chronic coccidioidomycosis. Having shown that neutrophils from healthy donors responded strongly to *C*. *posadasii* particles, we explored the possibility that abnormalities in neutrophil behavior might be related to the susceptibility of some humans to chronic coccidioidal infection. We used our single-cell/single-target approach to compare the responses of neutrophils from healthy and chronically ill donors to contact with two different targets: *C*. *posadasii* endospores, and antibody-coated beads (Fig 10).

Neutrophils from coccidioidomycosis patients responded as strongly to *C*. *posadasii* endospores as those from healthy donors. Moreover, the time-dependent morphology of neutrophils during phagocytosis of *C*. *posadasii* endospores was indistinguishable between the two donor groups (Fig 10). Thus neutrophils from coccidioidomycosis patients appeared to behave normally during encounters with the pathogen that causes the disease.

However, the neutrophils from coccidioidomycosis patients exhibited a distinct behavior in interactions with purely antibody-coated targets. Unlike the control neutrophils, the patient cells formed a noticeable protrusion at the onset of phagocytosis of antibody-coated beads (Fig 10A). The resulting maximum push-out distances were significantly larger than the push-out generated by the control group (Fig 10B). The average maximum outward displacement of the beads was 0.56±0.35(SD) μm for neutrophils from patients with chronic coccidioidal infection, in contrast to 0.12±0.14(SD) μm for healthy donors (*p*-value < 0.0001). A significantly longer engulfment time of antibody-coated beads by neutrophils from patients (*p*-value < 0.0001) was consistent with the larger push-out distance (Fig 10B). We also observed a moderate increase in the speed at which patient cells internalized antibody-coated beads (statistically significant with *p*-value of ~0.004).

A similar increase in the push-out distance of antibody-coated beads has been reported for neutrophils from healthy donors after treatment with actin inhibitors (latrunculin A or cytochalasin D) [43]. Generally, variations of the push-out distance have been attributed to differences in structural linkages between engaged cell-surface receptors and the cytoskeleton [33, 42]. Because the push-out distances of *C*. *posadasii* endospores did not vary between neutrophils from healthy donors and patients (unlike those of beads), we speculated that the distinct response of patient neutrophils to antibody-coated beads resulted from modulation of the interactions between Fcγ receptors and immobilized IgGs. Using flow cytometry we counted the Fcγ receptors displayed on the surfaces of control and patient neutrophils and found no significant differences, ruling out the possibility that the copy numbers of these receptors might have varied between the two donor groups (data not shown). Therefore, the observed push-out of antibody-coated beads by patient neutrophils suggests that serum IgGs and/or downstream processes might be affected by the chronic infection (and/or the antifungal drug regimens of the patients).

In summary, while the observed differences in the response to antibody-coated beads are difficult to interpret (but serve as an example of the discriminatory power of our single-cell approach), the main outcome of this section is that neutrophils from chronically ill patients do not exhibit detectable abnormalities during encounters with *C*. *posadasii* (by our assays).

## Discussion

There are intriguing clinical differences between the immune-cell responses to infections caused by different fungi. For example, neutrophils generally appear to play a larger role in aspergillosis or candidiasis than in coccidioidomycosis or cryptococcosis [55]. A detailed understanding of the mechanistic origins of such differences, and of microbial strategies to evade recognition, is an essential prerequisite of informed diagnosis and early treatment of infections. Central to such an understanding are the cause-effect sequences—or mechanisms—by which innate immune cells recognize microbes. The efficiency of this recognition can vary dramatically depending on the host species [29, 30] and on both the type of immune cell as well as the type of microbe [27, 39].

To examine the cause-effect relationships that underlie the recognition of *C*. *posadasii*, we dissect the response of single host cells into separable processes, such as target recognition from a distance (chemotaxis), capture and sustained affixation of target particles (adhesion), engulfment of the particles (phagocytosis), and their "post-processing" inside phagosomes. Our experimental approach enables us to inspect—without interference from cell-substrate interactions—the time courses of chemotaxis, adhesion, and phagocytosis, as well as their interplay, for individual pairs of cell and target in unprecedented detail. On the other hand, because our approach is less suited to study comparatively slow post-processing functions, the present analysis does not address the phagosomal biochemistry, bearing in mind though that a fraction of *C*. *posadasii* particles seem to be able to survive, or might even benefit from, internalization by immune cells [18, 20, 22, 56, 57].

Our results leave little doubt that under the used conditions, human neutrophils typically are well able to recognize and attack the surfaces of opsonized *C*. *posadasii* endospores and spherules from a short distance and upon contact. Moreover, the principal neutrophil behavior was similar during encounters with these two types of *C*. *posadasii* particle. Although the cell response to *C*. *posadasii* spherules exhibited distinct morphological features (such as wider chemotactic pseudopods, or thinly spread cell shapes during frustrated phagocytosis), these variations can satisfactorily be explained by the much larger size of the spherules and do not require the invocation of qualitatively different biochemical recognition paths. Further, the response of neutrophils from patients with chronic coccidioidomycosis to *C*. *posadasii* endospores was not significantly compromised or enhanced in comparison with the response of neutrophils from healthy donors.

Our results on the straightforward recognition of *C*. *posadasii* spherules by human neutrophils conflict with an earlier bulk study [22] that reported a low number of attachments between neutrophils and spherules. We note though that this low number was not necessarily due to a lack of recognition but could have several alternative explanations. For example, we have demonstrated in Figs 5 and 6 how a strong neutrophil response leads to frustrated phagocytosis of *C*. *posadasii* spherules by neutrophils within a few minutes. In this situation, the spread-out cells are under unusually high stress, as manifested by the enormous increase of their surface area as well as the elevated cortical tension (Fig 8). Such stress is likely to accelerate neutrophil lysis, which might explain the low number of (remaining) neutrophils observed in contact with spherules after a 3-hour cell-target incubation period [22]. Furthermore, a shaker (as used in the earlier study) might not provide optimal mixing of neutrophils and the much heavier spherules, thus affecting the frequency of cell-target encounters and biasing recognition.

Our integrative study underscores the physiological significance of chemotaxis and adhesion, both of which have long been overshadowed by a predominant focus on phagocytosis and killing of microbes. Such an imbalance seems hardly justified if one recalls that complement plays a key role in fungal recognition [49], and that the main functions of complement during this recognition are *(i)* the recruitment of immune cells by chemotaxis [58], and *(ii)* the sustainment of cell-target adhesion [59]. Indeed, extracts from different *C*. *immitis* phases were found to induce serum-dependent chemotactic activity in neutrophils [60]. Our results (Fig 3) provide the first direct evidence that both intact endospores as well as intact spherules elicit chemotaxis by nearby neutrophils. This chemotaxis is suppressed in the absence of serum or in the presence of heat-treated serum, implying that it is indeed mediated by complement.

The strong response of human neutrophils to close encounters with *C*. *posadasii* prompted us to conduct a series of comparative experiments with other fungal pathogens, i.e., *C*. *albicans*, *C*. *neoformans*, *A*. *fumigatus*, and *R*. *oryzae*, as well as with zymosan and IgG-coated beads. The results clearly demonstrated that fungal recognition does not follow a single mechanistic route. Furthermore, during all inspected stages of target recognition, the human neutrophil response to *C*. *posadasii* was one of the strongest observed antifungal responses, incongruent with the low neutrophil involvement in clinical manifestations of coccidioidomycosis. This baffling discrepancy shows that the aptitude of neutrophils to recognize nearby microbes cannot be taken as a predictor of the cells' actual involvement in the respective host responses to infection. We hence conclude that it is the *facilitation* of close encounters between cells and targets through mobilization and recruitment of neutrophils that plays a decisive role in determining the ultimate extent of neutrophil involvement in interactions with infectious fungi.

The juxtaposition of vigorous chemotaxis of neutrophils toward coccidioidal surfaces versus the lack of neutrophil mobilization in coccidioidomycosis thus elucidates a fundamental paradigm of the recognition of microbes, i.e., that intact immunotaxis comprises an intricate spatiotemporal hierarchy of distinct chemotactic processes. We established above that the particular chemotactic process shown in Fig 3 is complement-mediated and short-range. Chemoattractant anaphylatoxins like complement fragment C5a, once produced at microbial surfaces and released, are quickly metabolized by serum-based carboxypeptidases, and thus cannot recruit neutrophils over long distances. Our chemotaxis measurements determined that the range of this form of recruitment indeed extended only over ~1–2 cell diameters. Therefore, long-range mobilization and recruitment of innate immune cells require the production of cytokines by host cells that respond early to an infection. Why then should there be a need for complement-mediated chemotaxis at all? This question touches upon a long-overlooked mechanistic dilemma of cytokine-guided chemotaxis: newly recruited immune cells must be able to locate the pathogen itself rather than the host cells that produce cytokines. The mechanism of the short-range "redirection" of immune cells toward the actual pathogen employs the exquisite sensitivity of immune cells to anaphylatoxins produced at nearby microbial surfaces.

It seems unlikely that the remarkable similarity between the time-dependent neutrophil morphologies during interactions with *C*. *posadasii* endospores and zymosan particles is coincidental. Instead, we speculate that this phenotypic similarity reflects closely related, if not identical, mechanisms governing the early biochemical recognition of these fungal surfaces. Therefore, it seems sensible to draw parallels between the better-studied neutrophil response to zymosan and the recognition of *C*. *posadasii*. For example, the formation of chemotactic pseudopods toward zymosan in the presence of serum (such as in Fig 9) is primarily mediated by the anaphylatoxin C5a (unpublished data). It thus stands to reason that production of C5a at the surface of *C*. *posadasii* endospores and spherules is also the main cause of short-range neutrophil chemotaxis toward *C*. *posadasii* (as documented in Figs 3 and 9) [58]. Likewise, it seems reasonable to assume that similar ligand landscapes decorate the surfaces of serum-opsonized zymosan and *C*. *posadasii*, eliciting a common neutrophil response to direct contact with these targets. Human neutrophils lack the mannose receptor [61], and their expression level of the β-glucan receptor dectin-1 is too low to contribute significantly to the recognition of fungal targets [62] (in contrast to the importance of dectin-1 for the effective recognition of *C*. *posadasii* in mice [63–65]). In fact, it has been shown that quiescent human neutrophils do not constitutively express significant amounts of active surface receptors capable of recognizing unopsonized zymosan (unpublished data). Control experiments with unopsonized *C*. *posadasii* endospores and spherules confirmed that the same holds true for the bare surface of *C*. *posadasii* particles. Thus there can be little doubt that the initial recognition of *C*. *posadasii* by passive human neutrophils requires opsonins. Under physiological-like conditions, zymosan indeed has been shown to be densely coated with both C3b and IgG, and its recognition by neutrophils was found to be mediated primarily by the concerted action of complement receptor 3 (CR3, or Mac-1) and Fcγ receptors [34, 59]. Based on our above comparison, similar biochemical interactions are likely to dominate the recognition of *C*. *posadasii* particles upon contact.

## Conclusions

A successful innate immune-cell defense against coccidioidal infection encompasses an elaborate sequence of cross-disciplinary processes. Each ineffectual step in this sequence raises the survival chances of the pathogen. There is evidence that a fraction of *Coccidioides* arthroconidia and endospores can endure the harsh phagolysosomal environment, which probably explains the relatively slow clearance of coccidioidal infection by healthy individuals. (In fact, *Coccidioides* is known to be able to persist indefinitely in a latent form that can be reactivated following host immunosuppression [66, 67].) However, the mechanisms by which immune cells recognize *Coccidioides* in the first place remain poorly understood and have been the focus of this paper. We investigated these mechanisms by mimicking, visualizing, and analyzing one-on-one encounters between initially quiescent human neutrophils (from healthy donors and coccidioidomycosis patients) and *C*. *posadasii* endospores and spherules as well as other fungi and model pathogens. Based on dual-micropipette manipulation, we designed single-cell experiments that allowed us to separately assess the roles of chemotaxis, adhesion, and phagocytosis in neutrophil interactions with *C*. *posadasii* and other targets.

This integrative approach revealed that the human neutrophil responses to *C*. *posadasii*, *C*. *albicans*, and zymosan are alike and are much stronger than the responses to *A*. *fumigatus*, *R*. *oryzae*, and *C*. *neoformans*. There also are clear differences between the time-dependent behaviors of neutrophils encountering *C*. *posadasii* particles and purely antibody-coated microspheres. These and other observations highlight the critical role of complement (in cooperation with IgGs) in the initial short-range recognition of *C*. *posadasii* particles by human neutrophils.

Somewhat unexpectedly, the neutrophil responses during close encounters with various fungi do not necessarily correlate with the clinical involvement of neutrophils in the respective fungal infections. This led us to conclude that the long-range mobilization of neutrophils via cytokines determines the overall degree of neutrophil involvement in the host defense against infections, whereas a primary and vital function of complement-mediated, short-range chemotaxis is to guide immune cells toward nearby microbial surfaces. In view of the vigorous response of neutrophils to close encounters with *C*. *posadasii* particles, we speculate that avoidance of long-range neutrophil recruitment is a key evasive strategy of *C*. *posadasii*. Intriguingly, this speculation hints at the possibility of novel immuno-therapeutic strategies that might hold promise in battling coccidioidal and other infections in the future. One important milestone in the pursuit of such strategies is our evidence that neutrophils recognize not only endospores but also spherules of the fungus, in contrast to earlier reports [22].

## Materials and Methods

### Ethics statement

Written informed consent was obtained from all subjects. The Institutional Review Board of the University of California Davis approved the protocol covering this study.

### Human neutrophil isolation

Neutrophils were isolated from heparinized blood of healthy donors as described previously [32, 43]. Whole blood (5 mL) was placed on top of 4 mL PMN separation medium (Matrix/Thermo Fisher Scientific, Waltham, MA, USA) and centrifuged at 700 g for 30 min to separate polymorphonuclear cells from peripheral blood mononuclear cells. To remove remaining platelets, the recovered polymorphonuclear cell fraction was washed at 200 g for 10 min in Hanks’ balanced salt solution (HBSS, without calcium or magnesium; Sigma-Aldrich, St. Louis, MO, USA) containing 0.1% human serum albumin (HSA; Gemini, Sacramento, CA, USA). Finally, the cells were re-suspended in HBSS (without HSA) until use. Typically ~90% of recovered polymorphonuclear cells were neutrophils (as judged by optical microscopy).

### Preparation of fungal and model targets

As detailed below, the five fungal target species tested in this study were grown in routine laboratory media following specific protocols that are suitable and broadly used for the respective species. For example, the maintenance of *Coccidioides* spp. in the spherule/endospore stage requires strict control over both temperature and media conditions (growth in Converse broth media). Growth of molds such as *Aspergillus fumigatus* or *Rhizopus oryzae* in Converse media does not result in conidia for examination, but only mold forms. It is important to bear in mind that different media can result in different growth characteristics, which could have an effect on our quantitative assessment of the behavior of these fungi. However, we do not expect the differences in growth conditions to qualitatively alter the binary outcomes of our tests on whether or not the fungal particles are recognized by human neutrophils.

#### *Coccidioides posadasii* particles

Spherule-endospore (SE) cultures were prepared as previously described using the *C*. *posadasii* strain Silveira [68]. All work with potentially viable *C*. *posadasii* forms was performed under biosafety-level-3 conditions. In brief, stock cultures were maintained on 2XGYE agar slants at room temperature. For growth to the SE phase, arthroconidia were harvested from 4-week-old cultures by the spin bar technique of Sun and Huppert [15] and inoculated into 500 mL of modified Converse medium supplemented with 0.05% NZ-Amine (Sigma-Aldrich, St. Louis, MO) [69]. Cultures were incubated at 38°C, and 160 rpm, for up to 120 hours. Different morphogenic stages of the parasitic cycle were harvested by isolation at various times in their development and centrifugation conditions as previously described [70].

For single-cell experiments, spherules and endospores harvested at different time points were inactivated with thimerosal (1:10,000 final concentration), washed with endotoxin-free Cellgro PBS, pH 7.4 (Mediatech, Herndon, VA, USA), and stored at 4°C in phosphate-buffered saline (PBS) + thimerosal (1:10,000 concentration) until use [68].

#### *Candida albicans* cells

*C*. *albicans* was grown on Sabouraud dextrose agar plates (changed weekly) in an oven at 37°C.

#### *Cryptococcus neoformans* cells

*C*. *neoformans* (strain H99) was grown in YPD broth in a shaker water bath at 35°C with continuous moderate oscillation. Yeast suspensions were harvested at 24 hours and washed twice with 1X Phosphate Buffered Saline (Life Technologies, Grand Island, NY, USA).

#### *Aspergillus fumigatus* conidia

*A*. *fumigatus* (ATCC 204304) was grown on YPD agar slants in an incubator at 35°C until the formation of conidia (approximately 48 hours). Conidia were harvested by overlaying the slant with sterile media (RPMI 1640, with L-glutamine, without sodium bicarbonate, MP Biomedicals, Solon, OH, USA) containing 0.01% TWEEN 20 (Sigma-Aldrich, St. Louis, MO, USA). A conidium suspension was prepared by gently probing the colonies with the tip of a transfer pipette. After allowing large particles to settle for 3 to 5 minutes, the suspension was washed twice with media.

#### *Rhizopus oryzae* conidia

*R*. *oryzae* (ATCC 10404) was grown on YPD agar slants in an incubator at 35°C until the formation of conidia (approximately 72–96 hours), which were then harvested as described above for *A*. *fumigatus* conidia.

#### Zymosan particles

Stock zymosan particles (Sigma-Aldrich) were suspended in phosphate buffered saline (PBS; USB Corp., Cleveland, OH) at 2–10 mg/mL, boiled in a water bath for 30 min and centrifuged for 10 min at 400 g. The pellet was resuspended in PBS, and the boiling and washing steps were repeated twice more. Zymosan particles were stored at 4°C with 20 μM sodium azide until use.

#### Antibody-coated beads

Polystyrene microspheres were opsonized with antibody as described previously [32]. In short, 3 μm (nominal diameter) beads (Duke Scientific/Thermo Fisher Scientific, Waltham, MA) were incubated overnight at 4°C in PBS containing 10 mg/mL bovine serum albumin (BSA; Sigma-Aldrich). After three washes in PBS without BSA, the beads were incubated at room temperature with rabbit polyclonal anti-BSA antibody (Sigma-Aldrich) for 1 h. The beads were then washed three more times and re-suspended in PBS for storage at 4°C.

### Experiment chamber and micropipette manipulation

The preparation of micropipettes and of the experimental chamber were described in detail in previous publications [32, 38]. In short, micropipettes with an evenly broken, cylindrical tip of the desired inner diameter (here: ~2–3 μm) were routinely produced before each experiment. Each pipette was mounted on a motorized 3-axis manipulator. Custom-written software allowed us to control both pipettes with a single game joystick.

The experiment chamber was created by trapping buffer solution (HBSS with calcium and magnesium, usually supplemented with 10% autologous serum) between two horizontal microscope coverslips (cf. Fig 2A). To ensure quiescence of isolated neutrophils in the chamber, the bottom coverslip was passivated by covalent attachment of 2-[methoxy (polyethyleneoxy) propyl] trichlorosilane (PEG-silane, Gelest, Morrisville, PA) as described previously [41]. The pipettes accessed the interior of this chamber through its two open sides. Vertically movable water reservoirs controlled the pipette-aspiration pressure. The pressure of the cell-holding pipette was monitored in real time by measuring the height difference between the reservoir connected to the pipette and a pre-zeroed reference reservoir.

For phagocytosis experiments, a small volume of cell suspension (~5 μL) was introduced into the experiment chamber containing HBSS with calcium and magnesium with 10% autologous serum that had been heat-treated (~52–56°C for 45 min) and filtered. The preparation of chemotaxis experiments was similar, except that the chamber buffer contained normal (untreated) autologous serum.

### Single-live-cell/single-target experiments

All single-cell experiments were carried out at room temperature on a Zeiss AxioObserver inverted microscope (63× objectives with NA of 0.8 or 0.75). Small volumes of fungal particles or antibody-coated beads ("targets") were added to the cell suspension in the experiment chamber. For chemotaxis experiments, individual target particles and quiescent neutrophils were selected, aspirated with gentle pressure at the tips of opposing micropipettes, lifted above the chamber bottom, and positioned at the desired distance from each other (2.5–10 μm). After 1–2 min, or when a forming pseudopod reached a length of ≥2 μm, the target was relocated to a different position. For phagocytosis experiments, the approach was similar, but the target was immediately brought into gentle contact with the surface of the aspirated cell. Once adhesion occurred (usually immediately), the target was released from its pipette. In control experiments using BSA-coated beads (without antibody), no adhesion or phagocytosis was observed.

RM-series GigE Vision video cameras (JAI Inc.), Validyne pressure transducers (Validyne Engineering Corp.), and custom-written software were used to record video images of the chemotactic or phagocytic cell responses along with the pipette-aspiration pressure to computer hard disk at adjustable time intervals.

Another custom-written software application facilitated the later analysis of the recorded images, including the measurement of the target position (illustrated in Fig 7) and of the cell-projection length in the pipette. If needed, the cell-surface area and cortical tension were determined as described previously [43]. The size of the base of chemotactic pseudopods was estimated by measuring the width of this base in images such as shown in Fig 3. Assuming a circular cross section of the pseudopod base, the cross-sectional area was calculated. We report the size of the pseudopod base as a fraction of the original cell-surface area of the neutrophil, where the latter was determined by approximating the shape of the initially resting cell as a sphere.

### Cell staining

Samples of blood cells (after neutrophil enrichment, Fig 1, and after incubation with *C*. *posadasii* spherules, Fig 6) were fixed with formalin, deposited onto microscope slides by cyto-centrifugation (StatSpin CytoFuge, Iris Sample Processing, Inc., Westwood, MA), and stained with hematoxylin and eosin (H&E stain).

### Statistical analysis

Where indicated, significance of the difference between means was established by ANOVA using JMP (SAS Institute Inc.) or Origin software (OriginLab Corp., Northampton, MA). Only two-sample comparisons of means were performed in this study, in which case ANOVA is equivalent to a two-sample t-test.

## Supporting Information

S1 Video Pure chemotaxis of human neutrophils to endospores of *Coccidioides posadasii*. This video combines 3 examples of chemotaxis experiments (in the presence of untreated, autologous serum) in which a neutrophil sensed the presence and location of a *C*. *posadasii* endospore from a distance, before making physical contact with the fungus. The cell was held above the chamber bottom at the tip of a micropipette by gentle suction, allowing us to assess its chemotactic behavior without interference from cell-substrate interactions. The experiments demonstrate the aptitude of neutrophils to act as a highly sensitive biosensor of otherwise hard-to-detect, minuscule amounts of chemoattractants, such as the anaphylatoxin C5a. The endospore was eventually handed over to the neutrophil, resulting in firm adhesion and quick phagocytosis. (All 3 videos are sped up about 90 times.)
(MOV)

S2 Video Phagocytosis of *C*. *posadasii* endospores by human neutrophils. This video combines 3 movies of phagocytosis experiments in which a *C*. *posadasii* endospore was brought into contact with a pipette-held neutrophil and released (in the presence of heat-treated, autologous serum). Brief cell-target contact sufficed to establish adhesion and initiate phagocytosis. At the onset of target uptake, all neutrophils formed a protrusive "pedestal" that at first displaced the endospore outwards (i.e., away from the main cell body), which we have identified as a distinctive, target-specific morphological feature of neutrophil interaction with fungal particles (in heat-treated serum). (All 3 videos are sped up about 90 times.)
(MOV)

S3 Video Frustrated phagocytosis of an immature *C*. *posadasii* spherule by a human neutrophil. After first verifying that chemotaxis was impaired by heat treatment of the used serum, the immature spherule was brought into contact with the neutrophil, leading to quick recognition, firm adhesion, and frustrated phagocytosis. (The video is sped up about 90 times. The whole experiment took ~48 minutes.)
(MOV)

S4 Video Frustrated phagocytosis of a mature *C*. *posadasii* spherule by a human neutrophil. After first verifying that chemotaxis was impaired by heat treatment of the used serum, the mature spherule was brought into contact with the neutrophil, leading to quick recognition, firm adhesion, and frustrated phagocytosis. (The video is sped up about 90 times. The whole experiment took ~40 minutes.)
(MOV)

S5 Video Frustrated phagocytosis of an immature *C*. *posadasii* spherule by two human neutrophils. Shortly after an immature spherule was manipulated into contact with a first neutrophil, a second neutrophil touched and adhered to the spherule by chance. Both cells proceeded to spread over the spherule surface as much as possible. (The video is sped up about 150 times. The experiment took ~21 minutes.)
(MOV)

S6 Video Frustrated phagocytosis of a mature *C*. *posadasii* spherule by three human neutrophils. Using micropipettes, three neutrophils were sequentially brought into contact with the same spherule and proceed to attack it. (The video is sped up about 90 times. The experiment took ~14 minutes.)
(MOV)

## References

[1] Kupferschmidt K. Mycology. Attack of the clones. Science. 2012;337(6095):636–8. Epub 2012/08/11. 10.1126/science.337.6095.636 . 22879478

[2] Brown GD, Denning DW, Gow NA, Levitz SM, Netea MG, White TC. Hidden killers: human fungal infections. Science translational medicine. 2012;4(165):165rv13. 10.1126/scitranslmed.3004404 . 23253612

[3] Brown GD, Denning DW, Levitz SM. Tackling human fungal infections. Science. 2012;336(6082):647 10.1126/science.1222236 . 22582229

[4] Fisher MC, Henk DA, Briggs CJ, Brownstein JS, Madoff LC, McCraw SL, et al Emerging fungal threats to animal, plant and ecosystem health. Nature. 2012;484(7393):186–94. Epub 2012/04/14. 10.1038/nature10947 . 22498624 PMC3821985

[5] Galgiani JN, Ampel NM, Blair JE, Catanzaro A, Johnson RH, Stevens DA, et al Coccidioidomycosis. Clin Infect Dis. 2005;41(9):1217–23. Epub 2005/10/06. 10.1086/496991 . 16206093

[6] Kirkland TN, Fierer J. Coccidioidomycosis: a reemerging infectious disease. Emerging infectious diseases. 1996;2(3):192–9. Epub 1996/07/01. 10.3201/eid0203.960305 8903229 PMC2626789

[7] Marsden-Haug N, Goldoft M, Ralston C, Limaye AP, Chua J, Hill H, et al Coccidioidomycosis acquired in Washington State. Clin Infect Dis. 2013;56(6):847–50. Epub 2012/12/12. 10.1093/cid/cis1028 . 23223598

[8] Brown J, Benedict K, Park BJ, Thompson GR 3rd. Coccidioidomycosis: epidemiology. Clinical epidemiology. 2013;5:185–97. Epub 2013/07/12. 10.2147/CLEP.S34434 23843703 PMC3702223

[9] CDC-U.S. Increase in reported coccidioidomycosis—United States, 1998–2011. MMWR. 2013;62(12):217–21. 23535687 PMC4605002

[10] Sunenshine RH, Anderson S, Erhart L, Vossbrink A, Kelly PC, Engelthaler D, et al Public health surveillance for coccidioidomycosis in Arizona. Ann N Y Acad Sci. 2007;1111:96–102. Epub 2007/05/22. 10.1196/annals.1406.045 . 17513465

[11] Vugia DJ, Wheeler C, Cummings KC, Karon A. Increase in Coccidioidomycosis—California, 2000–2007. MMWR Morb Mortal Wkly Rep. 2009;58(5):105–9. Epub 2009/02/14 . 19214158

[12] Thompson GR 3rd. Pulmonary coccidioidomycosis. Seminars in respiratory and critical care medicine. 2011;32(6):754–63. Epub 2011/12/15. 10.1055/s-0031-1295723 . 22167403

[13] Einstein HE, Johnson RH. Coccidioidomycosis: new aspects of epidemiology and therapy. Clin Infect Dis. 1993;16(3):349–54. Epub 1993/03/01 . 8452945 10.1093/clind/16.3.349

[14] Chosewood LC, Wilson DE, Centers for Disease Control and Prevention (U.S.), National Institutes of Health (U.S.). Biosafety in microbiological and biomedical laboratories 5th ed. Washington, D.C.: U.S. Dept. of Health and Human Services, Public Health Service, Centers for Disease Control and Prevention, National Institutes of Health; 2009 xxii, 415 p. p.

[15] Sun SH, Huppert M. A cytological study of morphogenesis in *Coccidioides immitis* . Sabouraudia. 1976;14(2):185–98. Epub 1976/07/01 . 959944

[16] Huppert M, Sun SH, Harrison JL. Morphogenesis throughout saprobic and parasitic cycles of *Coccidioides immitis* . Mycopathologia. 1982;78(2):107–22. Epub 1982/05/22 . 7099241 10.1007/BF00442634

[17] Kolivras KN, Comrie AC. Modeling valley fever (coccidioidomycosis) incidence on the basis of climate conditions. International journal of biometeorology. 2003;47(2):87–101. Epub 2003/03/21. 10.1007/s00484-002-0155-x . 12647095

[18] Beaman L, Holmberg CA. In vitro response of alveolar macrophages to infection with *Coccidioides immitis* . Infect Immun. 1980;28(2):594–600. Epub 1980/05/01 6772563 10.1128/iai.28.2.594-600.1980 PMC550975

[19] Beaman L, Holmberg CA. Interaction of nonhuman primate peripheral blood leukocytes and *Coccidioides immitis* in vitro. Infect Immun. 1980;29(3):1200–1. Epub 1980/09/01 6776061 10.1128/iai.29.3.1200-1201.1980 PMC551260

[20] Drutz DJ, Huppert M. Coccidioidomycosis: factors affecting the host-parasite interaction. J Infect Dis. 1983;147(3):372–90. Epub 1983/03/01 . 6300253 10.1093/infdis/147.3.372

[21] Segal GP, Lehrer RI, Selsted ME. In vitro effect of phagocyte cationic peptides on *Coccidioides immitis* . J Infect Dis. 1985;151(5):890–4. Epub 1985/05/01 . 3989323 10.1093/infdis/151.5.890

[22] Frey CL, Drutz DJ. Influence of fungal surface components on the interaction of *Coccidioides immitis* with polymorphonuclear neutrophils. J Infect Dis. 1986;153(5):933–43 . 3701107 10.1093/infdis/153.5.933

[23] Galgiani JN. Inhibition of different phases of *Coccidioides immitis* by human neutrophils or hydrogen peroxide. J Infect Dis. 1986;153(2):217–22. Epub 1986/02/01 . 3944479 10.1093/infdis/153.2.217

[24] Richards JO, Ampel NM, Galgiani JN, Lake DF. Dendritic cells pulsed with *Coccidioides immitis* lysate induce antigen-specific naive T cell activation. J Infect Dis. 2001;184(9):1220–4. Epub 2001/10/13. 10.1086/323664 . 11598850

[25] Richards JO, Ampel NM, Lake DF. Reversal of coccidioidal anergy in vitro by dendritic cells from patients with disseminated coccidioidomycosis. J Immunol. 2002;169(4):2020–5. Epub 2002/08/08 . 12165528 10.4049/jimmunol.169.4.2020

[26] Cox RA, Magee DM. Coccidioidomycosis: host response and vaccine development. Clin Microbiol Rev. 2004;17(4):804–39 . 15489350 10.1128/CMR.17.4.804-839.2004 PMC523560

[27] Behnsen J, Narang P, Hasenberg M, Gunzer F, Bilitewski U, Klippel N, et al Environmental Dimensionality Controls the Interaction of Phagocytes with the Pathogenic Fungi *Aspergillus fumigatus* and *Candida albicans* PLoS Pathog. 2007;3(2):e13 10.1371/journal.ppat.0030013 17274685 PMC1790725

[28] Nathan C. Neutrophils and immunity: challenges and opportunities. Nat Rev Immunol. 2006;6(3):173–82. http://www.nature.com/nri/journal/v6/n3/suppinfo/nri1785_S1.html. 16498448 10.1038/nri1785

[29] Seok J, Warren HS, Cuenca AG, Mindrinos MN, Baker HV, Xu W, et al. Genomic responses in mouse models poorly mimic human inflammatory diseases. Proceedings of the National Academy of Sciences. 2013. 10.1073/pnas.1222878110 PMC3587220 23401516

[30] Mestas J, Hughes CCW. Of Mice and Not Men: Differences between Mouse and Human Immunology. The Journal of Immunology. 2004;172(5):2731–8. 14978070 10.4049/jimmunol.172.5.2731

[31] McDonald JU, Cortini A, Rosas M, Fossati-Jimack L, Ling GS, Lewis KJ, et al In vivo functional analysis and genetic modification of in vitro-derived mouse neutrophils. The FASEB Journal. 2011;25(6):1972–82. 10.1096/fj.10-178517 21368104

[32] Herant M, Heinrich V, Dembo M. Mechanics of neutrophil phagocytosis: behavior of the cortical tension. J Cell Sci. 2005;118(9):1789–97 15827090 10.1242/jcs.02275

[33] Heinrich V, Lee C-Y. Blurred line between chemotactic chase and phagocytic consumption: an immunophysical single-cell perspective. J Cell Sci. 2011;124(18):3041–51. 10.1242/jcs.086413 21914817 PMC3172184

[34] Mankovich AR, Lee CY, Heinrich V. Differential effects of serum heat treatment on chemotaxis and phagocytosis by human neutrophils. PLoS One. 2013;8(1):e54735 Epub 2013/01/26. doi: 10.1371/journal.pone.0054735 PONE-D-12-26803 [pii] 23349959 10.1371/journal.pone.0054735 PMC3551912

[35] Shubitz LF. Comparative aspects of coccidioidomycosis in animals and humans. Ann N Y Acad Sci. 2007;1111:395–403. 10.1196/annals.1406.007 . 17332082

[36] Graupmann-Kuzma A, Valentine BA, Shubitz LF, Dial SM, Watrous B, Tornquist SJ. Coccidioidomycosis in dogs and cats: a review. J Am Anim Hosp Assoc. 2008;44(5):226–35. 10.5326/0440226 . 18762558

[37] Herant M, Heinrich V, Dembo M. Mechanics of neutrophil phagocytosis: experiments and quantitative models. J Cell Sci. 2006;119:1903–13. PubMed Central PMCID: PMC16636075. 16636075 10.1242/jcs.02876

[38] Heinrich V, Rawicz W. Automated, high-resolution micropipet aspiration reveals new insight into the physical properties of fluid membranes. Langmuir. 2005 ; 21(5):1962–71 10.1021/la047801q 15723496

[39] Wangdi T, Lee C-Y, Spees AM, Yu C, Kingsbury DD, Winter SE, et al The Vi Capsular Polysaccharide Enables *Salmonella enterica* Serovar Typhi to Evade Microbe-Guided Neutrophil Chemotaxis. PLoS Pathog. 2014;10(8):e1004306 10.1371/journal.ppat.1004306 25101794 PMC4125291

[40] Swaney KF, Huang CH, Devreotes PN. Eukaryotic chemotaxis: a network of signaling pathways controls motility, directional sensing, and polarity. Annu Rev Biophys. 2010;39:265–89. Epub 2010/03/03. 10.1146/annurev.biophys.093008.131228 . 20192768 PMC4364543

[41] Lam J, Herant M, Dembo M, Heinrich V. Baseline mechanical characterization of J774 macrophages. Biophys J. 2009;96(1):248–54. PubMed S0006-3495(08)00006-4 10.1529/biophysj.108.139154 18835898 PMC2710052

[42] Herant M, Lee C-Y, Dembo M, Heinrich V. Protrusive push versus enveloping embrace: Computational model of phagocytosis predicts key regulatory role of cytoskeletal membrane anchors. PLoS Comput Biol. 2011;7(1):e1001068 10.1371/journal.pcbi.1001068 PubMed Central PMCID: PMCPMC3029235. 21298079 PMC3029235

[43] Lee C-Y, Herant M, Heinrich V. Target-specific mechanics of phagocytosis: protrusive neutrophil response to zymosan differs from the uptake of antibody-tagged pathogens. J Cell Sci. 2011;124(7):1106–14. 10.1242/jcs.078592 PubMed Central PMCID: PMCPMC3056606 21385838 PMC3056606

[44] Nathan CF. Neutrophil activation on biological surfaces. Massive secretion of hydrogen peroxide in response to products of macrophages and lymphocytes. The Journal of Clinical Investigation. 1987;80(6):1550–60. 10.1172/JCI113241 2445780 PMC442423

[45] Pillemer L, Ecker EE. Anticomplementary factor in fresh yeast. J Biol Chem. 1941;137(1):139–42.

[46] Ecker EE, Pillemer L, Seifter S. Immunochemical Studies on Human Serum: I. Human Complement and Its Components. J Immunol. 1943;47(3):181–93.

[47] Di Carlo FJ, Fiore JV. On the composition of zymosan. Science. 1958;127(3301):756–7. Epub 1958/04/04 . 13543326 10.1126/science.127.3301.756-a

[48] O'Meara TR, Alspaugh JA. The *Cryptococcus neoformans* Capsule: a Sword and a Shield. Clinical Microbiology Reviews. 2012;25(3):387–408. 10.1128/cmr.00001-12 22763631 PMC3416491

[49] Voelz K, May RC. Cryptococcal Interactions with the Host Immune System. Eukaryotic Cell. 2010;9(6):835–46. 10.1128/ec.00039-10 20382758 PMC2901644

[50] Del Poeta M. Role of Phagocytosis in the Virulence of *Cryptococcus neoformans* . Eukaryotic Cell. 2004;3(5):1067–75. 10.1128/ec.3.5.1067-1075.2004 15470235 PMC522596

[51] Kozel TR, Pfrommer GS, Guerlain AS, Highison BA, Highison GJ. Role of the capsule in phagocytosis of *Cryptococcus neoformans* . Rev Infect Dis. 1988;10 Suppl 2:S436–9 . 3055212 10.1093/cid/10.supplement_2.s436

[52] Kozel TR, Pfrommer GS, Redelman D. Activated neutrophils exhibit enhanced phagocytosis of *Cryptococcus neoformans* opsonized with normal human serum. Clin Exp Immunol. 1987;70(1):238–46 2961491 PMC1542204

[53] Latge JP. *Aspergillus fumigatus* and aspergillosis. Clin Microbiol Rev. 1999;12(2):310–50. Epub 1999/04/09 ; PubMed Central PMCID: PMCPmc88920. 10194462 10.1128/cmr.12.2.310 PMC88920

[54] Shevchenko MA, Bolkhovitina EL, Servuli EA, Sapozhnikov AM. Elimination of *Aspergillus fumigatus* conidia from the airways of mice with allergic airway inflammation. Respiratory research. 2013;14:78 Epub 2013/07/31. 10.1186/1465-9921-14-78 ; PubMed Central PMCID: PMCPmc3735401. 23890251 PMC3735401

[55] Bonnett CR, Cornish EJ, Harmsen AG, Burritt JB. Early neutrophil recruitment and aggregation in the murine lung inhibit germination of *Aspergillus fumigatus* Conidia. Infect Immun. 2006;74(12):6528–39. 10.1128/IAI.00909-06 16920786 PMC1698102

[56] Beaman L, Benjamini E, Pappagianis D. Role of lymphocytes in macrophage-induced killing of *Coccidioides immitis* in vitro. Infect Immun. 1981;34(2):347–53. Epub 1981/11/01 7309228 10.1128/iai.34.2.347-353.1981 PMC350872

[57] Galgiani JN, Hayden R, Payne CM. Leukocyte effects on the dimorphism of *Coccidioides immitis* . J Infect Dis. 1982;146(1):56–63. Epub 1982/07/01 . 7086205 10.1093/infdis/146.1.56

[58] Guo RF, Ward PA. Role of C5a in inflammatory responses. Annu Rev Immunol. 2005;23:821–52. Epub 2005/03/18. 10.1146/annurev.immunol.23.021704.115835 . 15771587

[59] Hed J, Stendahl O. Differences in the ingestion mechanisms of IgG and C3b particles in phagocytosis by neutrophils. Immunology. 1982;45(4):727–36. Epub 1982/04/01 6279489 PMC1555406

[60] Galgiani JN, Isenberg RA, Stevens DA. Chemotaxigenic activity of extracts from the mycelial and spherule phases of *Coccidioides immitis* for human polymorphonuclear leukocytes. Infect Immun. 1978;21(3):862–5. Epub 1978/09/01 711340 10.1128/iai.21.3.862-865.1978 PMC422076

[61] Pontow SE, Kery V, Stahl PD. Mannose receptor. Int Rev Cytol. 1992;137B:221–44. Epub 1992/01/01 . 1478821 10.1016/s0074-7696(08)62606-6

[62] van Bruggen R, Drewniak A, Jansen M, van Houdt M, Roos D, Chapel H, et al Complement receptor 3, not Dectin-1, is the major receptor on human neutrophils for beta-glucan-bearing particles. Mol Immunol. 2009;47(2–3):575–81. Epub 2009/10/09. S0161-5890(09)00720-2 [pii] 10.1016/j.molimm.2009.09.018 . 19811837

[63] Viriyakosol S, Fierer J, Brown GD, Kirkland TN. Innate Immunity to the Pathogenic Fungus *Coccidioides posadasii* Is Dependent on Toll-Like Receptor 2 and Dectin-1. Infection and Immunity. 2005;73(3):1553–60. 10.1128/iai.73.3.1553-1560.2005 15731053 PMC1064940

[64] del Pilar Jimenez AM, Viriyakosol S, Walls L, Datta SK, Kirkland T, Heinsbroek SE, et al Susceptibility to *Coccidioides* species in C57BL/6 mice is associated with expression of a truncated splice variant of Dectin-1 (Clec7a). Genes and immunity. 2008;9(4):338–48. Epub 2008/04/18. 10.1038/gene.2008.23 . 18418396 PMC3681288

[65] Drummond RA, Brown GD. The role of Dectin-1 in the host defence against fungal infections. Curr Opin Microbiol. 2011;14(4):392–9. 10.1016/j.mib.2011.07.001 21803640

[66] Raab SS, Silverman JF, Zimmerman KG. Fine-needle aspiration biopsy of pulmonary coccidiodomycosis. Spectrum of cytologic findings in 73 patients. American journal of clinical pathology. 1993;99(5):582–7. Epub 1993/05/01 . 8493952 10.1093/ajcp/99.5.582

[67] Miller MB, Hendren R, Gilligan PH. Posttransplantation disseminated coccidioidomycosis acquired from donor lungs. J Clin Microbiol. 2004;42(5):2347–9. Epub 2004/05/08 15131231 10.1128/JCM.42.5.2347-2349.2004 PMC404664

[68] Thompson GR 3rd, Lunetta JM, Johnson SM, Taylor S, Bays D, Cohen SH, et al Early treatment with fluconazole may abrogate the development of IgG antibodies in coccidioidomycosis. Clin Infect Dis. 2011;53(6):e20–4. Epub 2011/08/26. 10.1093/cid/cir466 . 21865185

[69] Converse JL, Besemer AR. Nutrition of the Parasitic Phase of *Coccidioides Immitis* in a Chemically Defined Liquid Medium. Journal of bacteriology. 1959;78(2):231–9. Epub 1959/08/01 16561837 10.1128/jb.78.2.231-239.1959 PMC290518

[70] Hung CY, Yu JJ, Lehmann PF, Cole GT. Cloning and expression of the gene which encodes a tube precipitin antigen and wall-associated beta-glucosidase of *Coccidioides immitis* . Infect Immun. 2001;69(4):2211–22. Epub 2001/03/20. 10.1128/IAI.69.4.2211-2222.2001 11254576 PMC98148

[71] Mirbod-Donovan F, Schaller R, Hung C-Y, Xue J, Reichard U, Cole GT. Urease Produced by *Coccidioides posadasii* Contributes to the Virulence of This Respiratory Pathogen. Infection and Immunity. 2006;74(1):504–15. 10.1128/iai.74.1.504-515.2006 16369007 PMC1346605

